# High-efficiency blue-emission crystalline organic light-emitting diodes sensitized by “hot exciton” fluorescent nanoaggregates

**DOI:** 10.1126/sciadv.add1757

**Published:** 2022-12-14

**Authors:** Jingjie Yang, Dehua Hu, Feng Zhu, Yuguang Ma, Donghang Yan

**Affiliations:** ^1^ State Key Laboratory of Polymer Physics and Chemistry, Changchun Institute of Applied Chemistry, Chinese Academy of Sciences, Changchun 130022, China.; ^2^ School of Applied Chemistry and Engineering, University of Science and Technology of China, Hefei 230026, China.; ^3^ State Key Laboratory of Luminescent Materials and Devices, South China University of Technology, Guangzhou 510640, China.

## Abstract

Sensitizing fluorescent materials is an effective way to maximally use excitons and obtain high-efficiency blue organic light-emitting diodes (OLEDs). However, it is a persistent challenge for present amorphous thin-film OLEDs to improve photon emission under low driving voltage, severely impeding the development of OLED technology. Here, we propose a novel OLED architecture consisting of a crystalline host matrix (CHM) and embedded “hot exciton” nanoaggregates (HENAs), which effectively sensitize blue dopant (D) emission. Owing to the advantages of the crystalline thin-film route, the device exhibits largely enhanced blue photon output [Commission International de L’Eclairage coordinates of (0.15, 0.17)], with a low turn-on/operation voltage of 2.5 V (at 1 cd/m^2^)/3.3 V (at 1000 cd/m^2^), an extremely low Joule heat loss ratio (7.8% at 1000 cd/m^2^), and a maximum external quantum efficiency (EQE) up to 9.14%. These areal photon output features have outperformed the present amorphous thin-film blue OLEDs with high EQE, demonstrating that the CHM-HENA-D OLED is promising for future OLEDs.

## Teaser

Organic crystalline thin films with “hot exciton” nanoaggregates make blue OLEDs stronger.

## INTRODUCTION

In past decades, blue fluorescent organic light-emitting diodes (BFOLEDs) have attracted tremendous attention in both academic and industry fields for their key roles in full-color displays (*1*, *2*). It is an essential requirement, yet a critical challenge, for the BFOLEDs to achieve high photon output with sufficient efficiency under low driving voltage. Because only 25% of electronically excited excitons are available for fluorescent emission because of spin statistics, the theoretical limit of the external quantum efficiency (EQE) of conventional BFOLEDs is approximately 5%, assuming a common light output coupling of 20% (*3*, *4*). To improve the utilization ratio of excitons, a strategy of sensitizers consisting of phosphorescence (*5*), thermally activated delayed fluorescence (TADF) (*6*, *7*), triplet-triplet annihilation (TTA) (*8*), and “hot exciton” materials (*9*) has been used to transfer more singlet/triplet excitons to the doped blue fluorescent emitters and improve device EQE. By applying phosphorescent and TADF sensitizers, BFOLEDs with high EQE have been achieved, as triplets are collected into sensitizers and delivered to blue emitters via energy transfer processes. However, the long lifetime of triplet excitons usually results in varying degrees of efficiency drop at the high luminance/current density level in their corresponding OLEDs (*10*). TTA can also effectively harness triplet excitons by generating one singlet exciton from the fusion of two triplet excitons, thereby providing a theoretical maximum internal quantum efficiency of 62.5% (*11*–*13*), which is lower than those of TADF- or phosphor-sensitized devices, though. Hot exciton materials have been proven to achieve almost 100% singlet exciton yield, owing to the efficient reverse intersystem crossing from high-lying triplet states to singlet excited states (i.e., T_2_ to S_1_, high-lying reverse intersystem crossing (hRISC)) and limited internal conversion (IC, i.e., T_2_ to T_1_) (*14*–*16*). The fast hRISC process of high energy excitons (hot excitons) can effectively suppress the accumulation of triplet excitons, realizing a promising device performance with a low efficiency roll-off. Therefore, the method of sensitizing emitters by hot exciton materials is expected to achieve a high yield of exciton utilization for ideal blue fluorescent emissions. On the other hand, amorphous thin-film forms are widely adopted in present high-efficiency OLEDs, rendering pinhole-free uniform organic layers, thereby avoiding phase separation that is considered to seriously jeopardize the device efficiency and emission performance. However, the disordered molecular arrangement in the amorphous thin films results in low carrier mobility (*17*), leading to intrinsically poor photon emissions under the low driving voltage of the present amorphous thin-film OLEDs (A-OLEDs). Crystalline organic materials have shown advantageous properties when they are applied in light-emitting devices; for instance, high mobility transport and aligned dipole orientation lead to low driving voltage and high photon output (*18*–*24*). Therefore, the application of crystalline thin films together with hot exciton materials potentially provides an effective route to tackle the disadvantages of amorphous thin-film routes and obtain high-performance BFOLEDs.

In this work, we report a new type of sensitized OLED consisting of a crystalline host matrix (CHM) and hot exciton nanoaggregates (HENAs) doped with blue fluorescent dopants (D). Taking advantages of the high carrier mobility from crystalline thin films and the effective collection of triplets from HENAs, a high-efficiency BFOLED with a low turn-on/operation voltage of 2.5 V (at 1 cd/m^2^)/3.3 V (at 1000 cd/m^2^) and a maximum EQE up to 9.14% is created. Compared to all reported blue A-OLEDs with high EQE values, the CHM-HENA-D OLED exhibits an extremely low ratio of series resistance Joule heat to input power and an overwhelming capability of blue photon emission at limited driving voltage, proving the superiority of the crystalline route in OLEDs.

## RESULTS

### Architecture and performance of CHM-HENA-D OLEDs

**Fig. 1.**
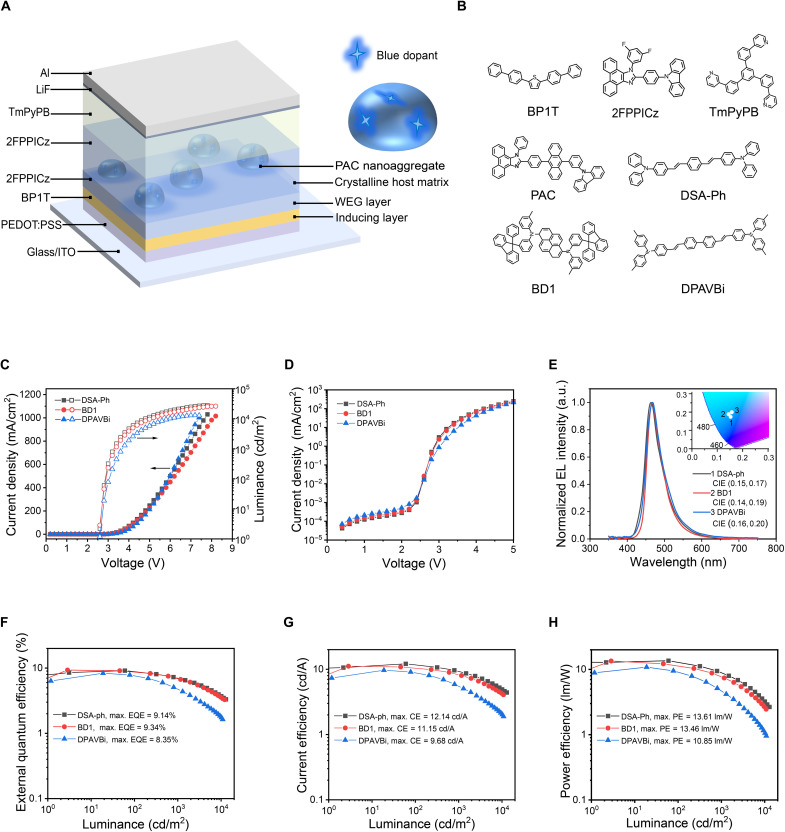
CHM-HENA-D OLEDs. (**A**) Schematic illustration of device structure. WEG, weak epitaxy growth. (**B**) Molecular formulas of materials. (**C**) Voltage-dependent current density and luminance. (**D**) Voltage-dependent simi-log current density. (**E**) EL spectrum at 1000 cd/m^2^ and the corresponding CIE of the devices with three different dopants. a.u., arbitrary units. (**F**) EQE-luminance curves of the devices. (**G**) Luminance-dependent CE characteristics. (**H**) Luminance-dependent PE characteristics.

The structure of the crystalline OLED (C-OLED) sensitized by the hot exciton fluorescent material 2-(4-(10-(3-(9H-carbazol-9-yl)phenyl) anthracen-9-yl)phenyl)-1-phenyl-1H-phenanthro[9,10-d] imidazole (PAC) (*15*) and their molecular structures are shown in Fig. 1 (A and B). The thin films were grown in a vacuum chamber with a base pressure of 10^−4^ Pa. An indium tin oxide (ITO) substrate and a 40-nm-thick poly(3,4-ethylenedioxythiophene):polystyrene sulfonate (PEDOT:PSS) layer act as the anode and hole injection layer, respectively. A 6-nm-thick 2,5-di([1,1′-biphenyl]-4-yl)thiophene (BP1T) crystalline thin film was deposited on PEDOT:PSS at 102°C, which not only transports holes but also blocks electrons, owing to its hole-transport ability and suitable energy levels in OLEDs. A 5-nm-thick layer of 2-(4-(9H-carbazol-9-yl)phenyl)-1-(3,5-difluorophenyl)-1H-phenanthro[9,10-d]imidazole (2FPPICz) was deposited on BP1T to form a crystalline epitaxy substrate layer, serving as the hole transport layer in this device (*24*). Then, a 20-nm-thick 2FPPICz CHM was grown together with the embedded PAC nanoaggregates within which blue fluorescent materials were doped, forming an emitting layer (EML). A 30-nm-thick amorphous thin film of 1,3,5-tri[(3-pyridyl)-phen-3-yl]benzene (TmPyPB), 1-nm-thick LiF, and 150-nm-thick Al were deposited, acting as the electron transport layer, electron injection layer, and cathode, respectively.

Three fluorescent materials, 1-4-di-[4-(*N*,*N*-diphenyl)amino]styryl-benzene (DSA-Ph), BD1 (*25*), and 4,4′-bis [4-(di-p-tolylamino) styryl] biphenyl (DPAVBi), are doped in PAC nanoaggregates to act as the blue emitters. These three CHM-HENA-D OLEDs with doping concentrations of 2 weight % (wt %) were fabricated and characterized (Fig. 1, C to H). Figure 1C shows the current density–voltage–luminance (*J-V-L*) curves of the devices. All devices show turn-on voltages of about 2.5 V. Simultaneously, these devices exhibited prominent characteristics in which the luminance and current density rise fast at extremely low driving voltage, having a luminance of 1000 cd/m^2^ at 3.3, 3.4, and 3.7 V, respectively. Electroluminescence (EL) spectra of the devices are shown in Fig. 1E. Their emission peaks are 464, 468, and 468 nm with a similar full width at half maximum of approximately 50 nm. Their corresponding Commission International de L’Eclairage (CIE) values are (0.15, 0.17), (0.14, 0.19), and (0.16, 0.20), respectively. Figure 1F demonstrates EQE curves as a function of luminance. The maximum EQEs are 9.14, 9.23, and 8.35%, and the EQE values are maintained at 7.20, 7.02, and 4.74% at 1000 cd/m^2^, respectively. Figure 1 (G and H) presents the current efficiency (CE) and power efficiency (PE) of the devices, achieving maximum PEs of 13.61, 13.46, and 10.85 lm W^−1^, respectively. The performance of these CHM-HENA-D OLEDs is compared to reported state-of-the-art blue A-OLEDs with high EQE in table S1. It is worth noting that the turn-on voltage and driving voltage (1000 cd/m^2^) of CHM-HENA-D OLEDs are the smallest among reported blue OLEDs with high EQE (CIE*_y_* ≤ 0.2).

### Preparation of CHM and nanoaggregates

**Fig. 2.**
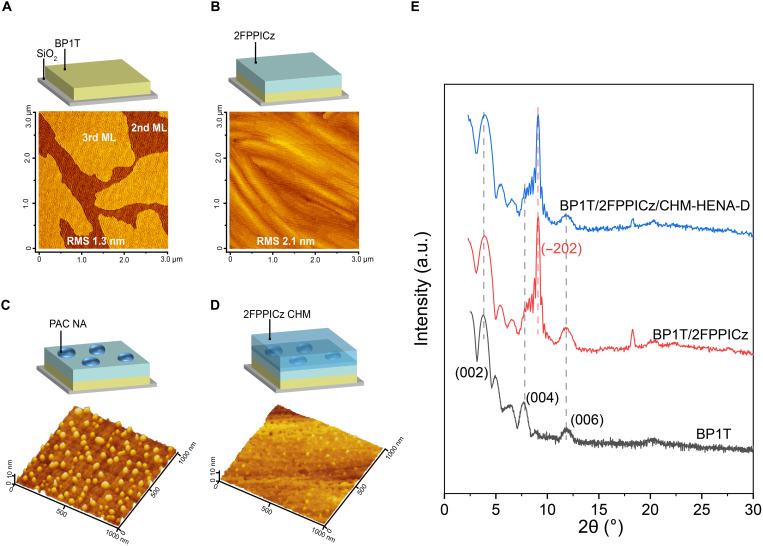
Preparation and characterization of crystalline thin film and nanoaggregates. (**A**) Schematic illustration and atomic force microscopy (AFM) image of BP1T crystalline thin film. (**B**) Schematic illustration and AFM image of the 2FPPICz crystalline epitaxy thin film. (**C**) Schematic illustration and three-dimensional (3D) AFM image of PAC nanoaggregates grown on the crystalline epitaxy thin film. (**D**) Schematic illustration and 3D AFM image of a 2FPPICz crystalline host containing PAC nanoaggregates. (**E**) Out-of-plane XRD patterns of BP1T crystalline thin film, 2FPPICz CHM, and 2FPPICz CHM containing PAC nanoaggregates with DSA-Ph 2 wt %.

To date, the efficient OLEDs consisting of organic nanostructures (e.g., nanocrystals and nanoaggregates) are rarely reported, as the phase separation seriously harms the device performance of host-dopant systems. It is a persistent challenge to use well-developed high-mobility organic semiconductor materials and nanostructures for fabricating high-efficiency OLEDs. Here, weak epitaxy growth (*26*, *27*), a convenient physical vapor deposition technique, was used for preparing crystalline thin films in the CHM-HENA-D OLEDs and, in parallel, for the investigation of thin-film structures. A 6-nm-thick BP1T smooth thin film [corresponding to monolayers (MLs) thicker than two] was first deposited on a Si/SiO_2_ substrate held at 102°C, acting as an inducing layer for the subsequent epitaxy growth of crystalline thin films. Figure 2A shows that the BP1T layer formed a continuous and large-sized crystalline thin film, which was composed of densely interconnected two MLs and the third ML crystalline domain growth on top. The root-mean-square (RMS) surface roughness is only 1.3 nm, demonstrating that the BP1T layer has a molecularly smooth surface. Then, the wide-bandgap fluorescent material 2FPPICz (*24*) was deposited onto the surface of the BP1T layer at 102°C (Fig. 2B) to form a 5-nm-thick crystalline epitaxy layer. The 2FPPICz crystalline layer maintains continuity with molecular-level smoothness (RMS of 2.2) and the stripe-like crystals within crystal domains are well connected, serving as the basis of the following 2FPPICz CHM. The hole and electron mobilities of 2FPPICz crystalline thin film in the direction perpendicular to the substrate are approximately 0.10 and 0.015 cm^2^ V^−1^ s^−1^, respectively, which were obtained by time-of-flight method (*24*). In low-mobility amorphous organic thin films, energetic and/or structure disorders are dominant in charge transport. Governed by the Poole-Frenkel mechanism, carrier mobilities of amorphous organic materials generally increase with the square root of electrical field strength (*17*). However, the hole and electron mobility values of the 2FPPICz crystalline thin film show a different characteristic that they decrease with increasing electrical field, suggesting that carrier transport in the 2FPPICz crystalline thin film does not follow the common mechanism in amorphous organic materials. These intrinsically high mobilities of crystalline materials are much larger than the mobility values of charge transport and EMLs in the present A-OLEDs (10^−8^ to 10^−2^ cm^2^ V^−1^ s^−1^) (*28*, *29*). Afterward, the hot exciton material PAC and blue dopant molecules (2 wt % to PAC) were codeposited on top of the 2FPPICz crystalline thin film at a substrate temperature of 102°C. Figure 2C and fig. S1 (A and B) show the morphology of nanoaggregates on the surface of the 2FPPICz crystalline thin film. It is observed that the nanoaggregates are homogeneously distributed on the surface of 2FPPICz crystalline film (fig. S1, A and B). The ratio of the vertical projection area of the nanoaggregates on the 2FPPICz layer was approximately 20% (fig. S1C). In addition, it can also be observed that the diameter and height of the nanoaggregates are 40 to 70 nm and 4 to 7 nm, respectively. There is a certain distance between the discrete nanoaggregates for the subsequent growth of the 2FPPICz crystalline thin film. Then, 2FPPICz was continuously deposited on the top of the 2FPPICz crystalline substrate with PAC nanoaggregates. The atomic force microscopy (AFM) image (Fig. 2D and fig. S1D) of the thin films shows that the 2FPPICz molecules can grow along with the areas where nanoaggregates did not cover to form a densely interconnected crystalline thin film, suggesting that nanoaggregates do not affect the integrity of the CHM. Therefore, it can be imagined that the nanoaggregates are embedded in the 2FPPICz crystalline framework and surrounded by highly ordered 2FPPICz molecules. Last, by repeating alternate growth of the nanoaggregates and 2FPPICz crystalline thin film for extra three times, an EML was formed. Figure S2 (A and B) shows the morphology images of the BP1T (6 nm)/2FPPICz (25 nm) with dimensions of 50 μm by 50 μm and 20 μm by 20 μm, respectively, and a large-size polycrystalline morphology with well-connected stripe-like crystals within crystal domains shows up. The morphology of the BP1T (6 nm)/2FPPICz (5 nm)/2FPPICz (20 nm) containing PAC nanoaggregates with DSA-Ph (2 wt %) [noted as BP1T (6 nm)/2FPPICz (5 nm)/CHM-HENA-D (20 nm)] thin film is similar to that of the 2FPPICz crystalline thin film, further demonstrating that nanoaggregates do not affect the growth and integrity of 2FPPICz CHM (fig. S2, C and D). The out-of-plane and in-plane x-ray diffraction (XRD) patterns of BP1T (6 nm) and BP1T (6 nm)/2FPPICz (25 nm) crystalline thin films are shown in Fig. 2E and fig. S3A, respectively, indicating their crystallinity nature, and are consistent with the previous report (*24*, *30*, *31*). The grazing incident wide-angle x-ray diffraction (GIWAXD) can be carried out to investigate the crystal structure (*18*). Figure S3B shows the GIWAXD images of 2FPPICz crystalline thin film with annotated (*hkl*) parameters according to (*31*), indicating that 2FPPICz CHM is composed of polycrystal with well-crystallinity nature. The out-of-plane XRD patterns of BP1T (6 nm)/2FPPICz (5 nm)/CHM-HENA-D (20 nm) are consistent with BP1T (6 nm)/2FPPICz (25 nm), and no other diffraction peak appears (Fig. 2E). This reveals that PAC nanoaggregates distributed in the 2FPPICz CHM are amorphous.

### Analysis of device mechanism

**Fig. 3.**
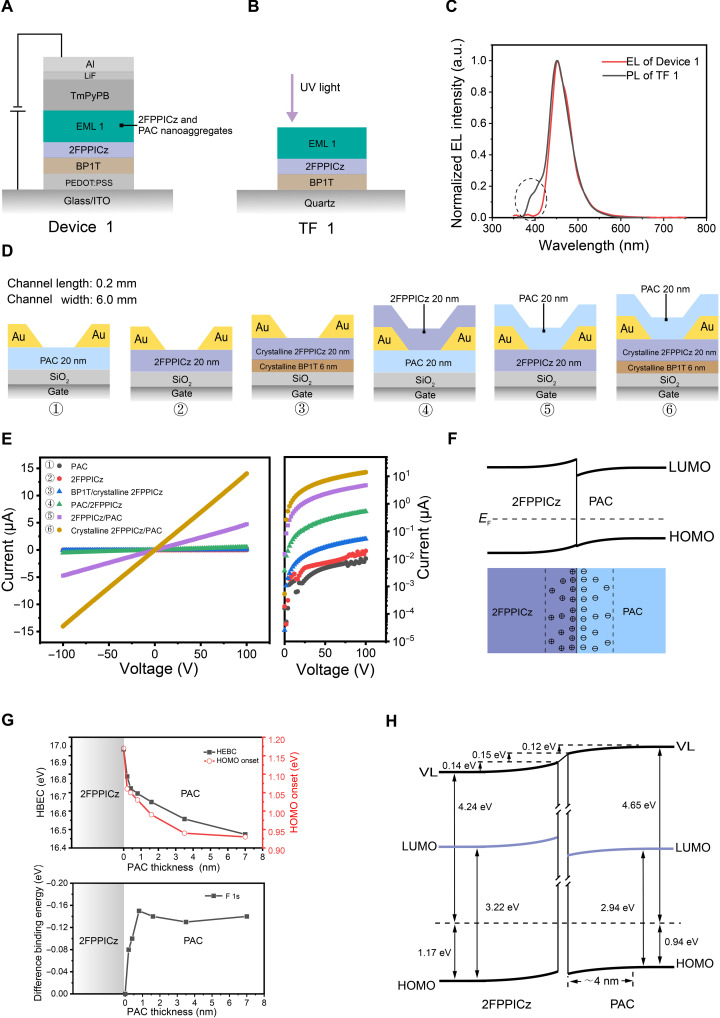
Analysis of device mechanism. (**A**) Structure of Device 1. (**B**) Structure of TF 1. UV, ultraviolet. (**C**) Comparison of the EL of Device 1 and the PL of TF 1. (**D**) Structures of in-plane transport devices. (**E**) Voltage- dependent current of in-plane transport devices. (**F**) Band bending diagram and space-charge accumulation diagram of the 2FPPICz/PAC heterojunction. (**G**) HBEC and HOMO onset positions in the UPS spectra and relative binding energy difference of F 1s core levels from 2FPPICz as a function of PAC thickness. (**H**) Schematic energy level diagram of the 2FPPICz crystalline film/PAC organic heterojunction.

To understand the emission mechanism of these CHM-HENA-D OLEDs, we first fabricated a reference Device 1, ITO/PEDOT:PSS (40 nm)/BP1T (6 nm)/2FPPICz (5 nm)/EML1 (20 nm)/TmPyPB (30 nm)/LiF (1 nm)/Al (150 nm) (Fig. 3A). Both BP1T and 2FPPICz layers are crystalline thin films, and the EML1 consists of a 2FPPICz CHM and PAC nanoaggregates without blue dopants. All of them were prepared by the method identical to the previous section. The performance of Device 1 is illustrated in fig. S4. Then, a reference thin-film structure TF 1, quartz substrate/BP1T (6 nm)/2FPPICz (5 nm)/EML1 (20 nm), was fabricated in the same way (Fig. 3B). The photoluminescence (PL) spectrum of TF 1 was measured at an excitation wavelength of 350 nm, where the absorption by 2FPPICz is dominant (fig. S5). It can be observed that there is a stronger 2FPPICz emission at wavelengths shorter than 400 nm in the PL spectra of TF 1, compared to the EL spectra of Device 1 (Fig. 3C). In Device 1, if excitons were formed in the region of 2FPPICz crystalline film, then the EL spectrum of 2FPPICz (around and below 400 nm) should also be observed, like the PL of TF 1. This reveals that, in Device 1, excitons were directly formed in PAC nanoaggregates instead of being transferred from 2FPPICz via an energy transfer process. It can be understood that the hole carriers injected from the anode pass through BP1T/2FPPICz and then encounter the electrons at PAC nanoaggregates. In addition, the turn-on voltage of Device 1 at 2.6 V is close to the photon energy of the PAC optical bandgap (2.74 eV, calculated from the PL emission maximum of PAC nanoaggregates at 452 nm), suggesting that carrier injection and transport took place without obstruction from energy barriers. To explore the mechanism of this unexpected phenomenon, we constructed another two reference OLEDs, Device 2 (only 2FPPICz used in EML) and Device 3 (only PAC used in EML) (fig. S6, A and B) with structures similar to that of Device 1. However, their EMLs consist of a 20-nm-thick 2FPPICz crystalline thin film (Device 2) and a 20-nm PAC amorphous thin film prepared at room temperature (Device 3). The turn-on voltage of Device 1 is only 2.6 V, evidently lower than the turn-on voltages of Device 2 (3.1 V) and Device 3 (2.9 V). Moreover, the current density of Device 1 (fig. S6C) is higher than that of Device 2 and Device 3 at the same driving voltage, indicating that Device 1 has a higher conductance and shows a quick turn-on characteristic. This result demonstrates that the coexistence of 2FPPICz and PAC is a key factor in high conductance and low turn-on voltage in these devices. The conductivity (σ) can be calculated as

σ=n×q×μ

where *n* is the charge concentration, *q* is the unit charge, and μ is the carrier mobility. Because of using the same crystalline thin film layer in Device 1 and Device 2, the reason why Device 1 has a higher conductance than that of Device 2 is due to the higher charge concentration in EML (2FPPICz CHM-HENA) of Device 1. Therefore, a charge transfer, which is a heterojunction effect between 2FPPICz and PAC, can take place when the PAC nanoaggregates are introduced into 2FPPICz CHM. To verify the heterojunction effect between 2FPPICz and PAC, several in-plane transport devices were fabricated, including ➀ amorphous PAC thin film (20 nm), ➁ amorphous 2FPPICz thin film (20 nm), ➂ BP1T crystalline thin film (6 nm)/2FPPICz crystalline thin film (20 nm), ➃ amorphous PAC thin film (20 nm)/amorphous 2FPPICz thin film (20 nm), ➄ amorphous 2FPPICz thin film (20 nm)/amorphous PAC thin film on top, and ➅ crystalline thin-film BP1T (6 nm)/2FPPICz (20 nm)/amorphous PAC thin film (20 nm), to investigate the hole-only transport property of the PAC and 2FPPICz thin film (Fig. 3D). No evident current was produced under electrical fields in Device ➀ (only PAC), Device ➁ (only 2FPPICz), or Device ➂. 2782;. However, Device ➄ and Device ➅ exhibited evident ohmic-like behavior (Fig. 3E). This result reveals that there is a heterojunction effect between PAC and 2FPPICz film, which generated a high conductive channel (*32*–*35*). Considering three facts: (i) injection of holes from Au electrodes into 2FPPICz is much easier than electrons as the Fermi level of Au is close to the highest occupied molecular orbital (HOMO) level of 2FPPICz, (ii) hole’s transport capability is superior to electron’s in 2FPPICz layer, and (iii) the conduction of Device ➅ is two orders higher than that of Device ➂, it can be inferred that the holes’ concentration and transport in 2FPPICz under the heterojunction effect is much enhanced compared to the situation of only 2FPPICz. It can be further inferred that, when 2FPPICz and PAC contact each other, charge transfer happens and electrons flow from 2FPPICz to PAC, causing a higher density of holes being accumulated in 2FPPICz. These results verify the heterojunction effect at the interface between 2FPPICz film and PAC, although they are not a strong donor-acceptor system. This charge transfer and carriers’accumulation take place as long as 2FPPICz and PAC are in contact, either in the direction perpendicular to the substrate or parallel to the substrate. In CHM-HENA OLED, PAC nanoaggregates are embedded in the 2FPPICz CHM and surrounded by highly ordered 2FPPICz molecules. Therefore, PAC nanoaggregates can contact 2FPPICz CHM, leading to a heterojunction effect in the interface between 2FPPICz CHM and PAC nanoaggregates. Electrons can flow from 2FPPICz CHM to PAC nanoaggregates, causing confined space-charges, i.e. holes and electrons, being accumulated in 2FPPICz CHM and PAC nanoaggregates, respectively, and, simultaneously, a band bending of 2FPPICz/PAC heterojunction interfaces formed (Fig. 3F). The result leads to the carrier concentration increased in the CHM-HENA region.

**Fig. 4.**
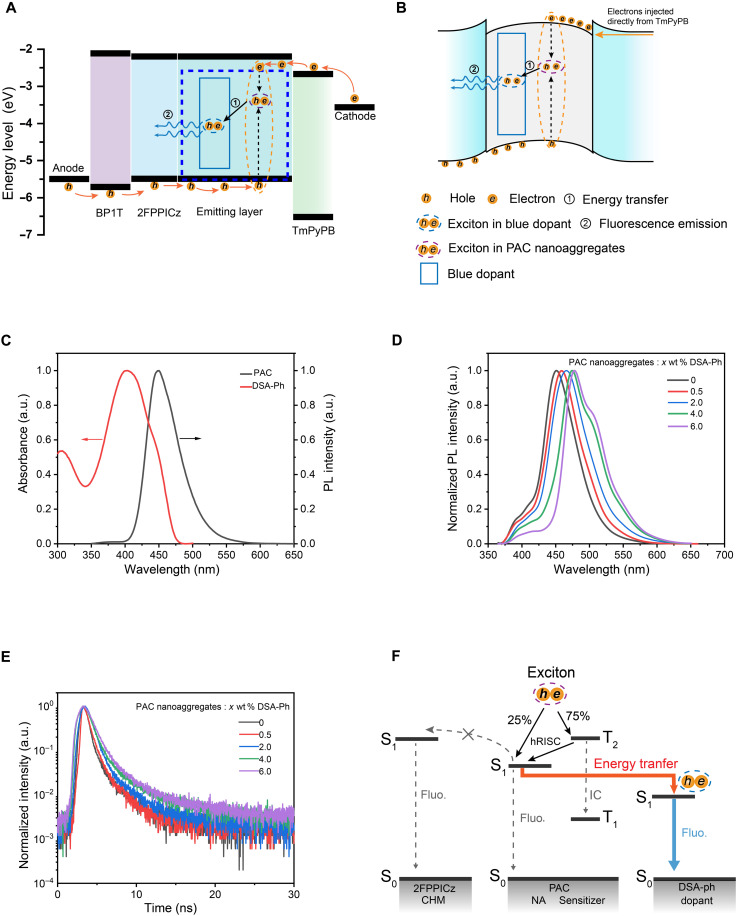
Mechanism of CHM-HENA-D OLED. (**A**) Schematic illustration of the energy-level diagram and working mechanism of the device. (**B**) Schematic diagram of excitons formed in PAC nanoaggregates. (**C**) Absorption spectra of DSA-Ph film and PL spectra of PAC film. (**D**) PL spectra of composite films consisting of PAC nanoaggregates: *x* wt % DSA-Ph (*x* = 0, 0.5, 2.0, 4.0, and 6.0). Excitation wavelength, 350 nm. (**E**) Transient decay curves of 465-nm emission bands of the composite films consisting of PAC nanoaggregates: *x* wt % DSA-Ph (*x* = 0, 0.5, 2.0, 4.0, and 6.0). Excitation wavelength, 350 nm. (**F**) Schematic diagram of the sensitization processes of PAC nanoaggregates.

To further verify the 2FPPICz/PAC heterojunction effect, ultraviolet (UV) photoemission spectroscopy (UPS) and x-ray photoemission spectroscopy (XPS) are performed to measure the BP1T (6 nm)/2FPPICz (5 nm) crystalline film/PAC (*x* nm) (*x* = 0.2, 0.4, 0.8, 1.6, 3.5, and 7). Figure S7A shows the evolution of UPS spectra for the 2FPPICz crystalline film/PAC system. It can be observed that the high–binding energy cutoff (HBEC) position of the films shifts to low energy as the PAC thickness increases, showing the shift in the vacuum level (VL) at the 2FPPICz crystalline film/PAC interface. The HOMO onset of the films shifts about 0.12 eV toward low energy with PAC thickness increasing, showing an energy level bending downward from bulk to interface in PAC. Figure S7B shows the evolution of N 1s core levels of films and F 1s core levels as PAC is deposited on the 2FPPICz crystalline film. The binding energy of N 1s shifts about 0.1 eV to low energy with the PAC thickness increasing, and the values of binding energy have hardly changed at PAC thickness of 3.5- and 7-nm area, which corresponds to the results of UPS. For the F 1s core levels that are only from the 2FPPICz crystalline film, the intensity of signal F 1s gradually decreases, and core levels show a total shift of about 0.14 eV to lower binding energy during the growth of PAC, demonstrating that 2FPPICz loses electrons and forms holes accumulation region near the heterojunction interface, causing an upward energy level bending from bulk to interface in 2FPPICz. The HBEC position, HOMO onset position, and difference binding energy core level of F 1s as a function of PAC thickness are shown in Fig. 3G. When the thickness of PAC exceeds 3.5 nm, the HOMO onset of the film maintains an almost same constant, estimating that the thickness of accumulated charge region in the PAC layer is about 4 nm. It can be easily understood that about 4-nm-thick electron accumulation region surrounds within the PAC nanoaggregates. The shift of HBEC is about 0.41 eV estimated from this charge region. When the PAC thickness exceeds 3.5 nm, the evident shift of HBEC position should be attributed to charging effects during UPS measurement. Therefore, these results indicate an upward energy level bending in the 2FPPICz crystalline film and a downward energy level bending in PAC, with the direction pointing from bulk to interface. The bending direction indicates that negative charges exist in PAC and positive charges exist in 2FPPICz crystalline film. Figure 3H depicts the schematic energy level diagram of the 2FPPICz crystalline film/PAC. We can conclude an energy level bending of 0.14 eV in 2FPPICz crystalline film, 0.12 eV in PAC, and an interface dipole of about 0.15 eV. The bandgap of 2FPPICz crystalline film and PAC is 3.22 and 2.94 eV, respectively, calculated from the absorption band edge (fig. S8). The lowest unoccupied molecular orbital (LUMO) values of 2FPPICz crystalline and PAC are calculated to be −2.19 and −2.65 eV based on the bandgap and the measured HOMO of 2FPPICz crystalline film and PAC, respectively. As a result, because of the low LUMO (−2.65 eV) and downward bending energy level of PAC from the bulk to the interface (about 0.12 eV), electrons are easily injected into PAC nanoaggregates from the LUMO of electrons transport layer TmPyPB (−2.74 eV) (*36*) without injection barrier. On the other hand, the top parts of PAC nanoaggregates expose out of the film surface (figs. S1D and S12A), greatly facilitating the direct injection of electrons from the electron transport layer into PAC nanoaggregates. Therefore, these two factors, a heterojunction effect in the interface between 2FPPICz CHM and PAC nanoaggregates and the barrier-free injection of electrons from the electrons transport layer to PAC nanoaggregates, are the reasons why Device 1 has a low turn-on and driving voltage compared to Device 2 and Device 3. Here, the emission process of the CHM-HENA-D OLEDs can be revealed. The energy level and mechanism of the device are depicted in Fig. 4A. The hole and electron carriers are injected from the anode and the cathode, respectively. The holes pass through BP1T/2FPPICz to EML and are transported along the 2FPPICz CHM until reaching PAC nanoaggregates. Meanwhile, the injected electrons pass through TmPyPB, transporting directly into PAC nanoaggregates under the electrical field. Then, the holes and electrons encounter each other to form excitons at PAC nanoaggregates (Fig. 4B). The hot exciton material PAC can effectively transfer electrically generated triplet excitons to singlets via the hRISC process according to the previous report (*15*). It is worth noting that, in (*15*), the hot exciton characteristics of PAC were proven in molecule form, indicating that the hRISC process of PAC shown in (*15*) can occur in the PAC nanoaggregates as they are both in amorphous form. Therefore, the blue fluorescent dye DSA-Ph with a high PL quantum yield (PLQY) is chosen as a dopant in PAC nanoaggregates to harvest excitons from PAC via an energy transfer process and achieve high-efficiency radiation emission. Then, the excitons are transferred to the S_1_ state of DSA-Ph from the S_1_ state of PAC via the energy transfer.

To verify the process of energy transfer between PAC nanoaggregates and DSA-Ph, several CHM-HENA-D films where PAC nanoaggregates doped with different concentrations of DSA-Ph, quartz substrate/BP1T (6 nm)/2FPPICz (5 nm)/2FPPICz crystalline matrix containing PAC nanoaggregates with *x* wt % DSA-Ph (20 nm) were prepared and tested. As shown in Fig. 4C, the absorption spectrum of DSA-Ph film partly overlaps with the PL spectrum of PAC film, indicating that an efficient energy transfer can be expected. PL spectra of CHM-HENA-D films with different concentrations of DSA-Ph (0, 0.5, 2, 4, and 6%) are shown in Fig. 4D. Compared to PAC nanoaggregates of CHM-HENA-D film (no dopant DSA-Ph) with an emission peak at 451 nm, other emitters with different concentrations of DSA-Ph show red-shifted with emission peaks of 458, 470, 474, and 479 nm (corresponding to 0.5, 2, 4, and 6%, respectively), demonstrating that a sufficient energy transfer occurs from PAC nanoaggregates to DSA-Ph as the concentration of DSA-Ph is increased. Furthermore, transient PL measurements of these films were performed at wavelength 470 nm where the emission of DSA-Ph is dominant. As shown in Fig. 4E, the emission lifetime of these films is increased as the concentration of DSA-Ph is increased in PAC nanoaggregates, also verifying the efficient energy transfer process. The relevant data are summarized in table S2. The energy transfer time scale from PAC nanoaggregates to DSA-Ph is 1.1 ps, which is gained by femtosecond transient absorption spectroscopy (fs-TA) measurements (fig. S9). These experimental results all prove the efficient energy transfer from PAC nanoaggregates to fluorescent dopant DAS-Ph. On the basis of the above conclusion, the energy transfer mechanism and sensitizing process in the CHM-HENA-D systems are depicted in Fig. 4F. In addition, the optical transition dipole moment vectors of DSA-Ph emitter in a CHM-HENA-D thin film are considered to be random and have no enhancement effect on the light outcoupling efficiency of the device by the angle- and polarization-dependent luminescence spectroscopy (ADPL) measurement (fig. S10A) (*37*, *38*). By using a similar device structure to CHM-HENA-D OLED, several reference devices were fabricated to estimate the light outcoupling efficiency of CHM-HENA-D OLED as approximately 20% (fig. S10, B to D). The exciton utilization efficiency (EUE) of CHM-HENA-D OELD can be calculated according to the equation

$$\mathrm{EQE}=(\gamma \times \mathrm{EUE}\times \Phi_{\mathrm{PL}})\times \eta_{\mathrm{out}}$$

where γ is the recombination efficiency of the injected electron and hole (generally identified as 100%), Φ_PL_ is the PLQY of the emitter, and η_out_ is the light outcoupling efficiency of the device. The Φ_PL_ of 2 wt % DSA-Ph doped in the CHM-HENA-D thin film is 0.81, and η_out_ of CHM-HENA-D device is estimated as approximately 20%. Therefore, the EUE of the CHM-HENA-D device is calculated as 56%. Several CHM-HENA-D OLEDs with different concentrations of DSA-Ph (0.5, 2, 4, 6, wt %) in PAC nanoaggregates were fabricated. The characteristics of these devices are demonstrated in fig. S11. It is noted that the current density was reduced as the concentration of the DSA-Ph was increased. This reveals that the dopant molecules can directly capture holes and electrons, increase resistance, and compete with PAC nanoaggregates in harvesting excitons when the doping concentration increases. The maximum EQE was also reduced as the concentration (>2 wt %) was increased, which can demonstrate that the competition exists, consistent with the fact that a low doping concentration was used in the previous reports (*9*, *39*). It was also observed that the EL of PAC (452-nm peak) appeared when 0.5 wt % was doped, indicating that the extremely low concentration of dopants is unable to completely collect excitons from PAC. Table 1 summarizes the performance comparison of DSA-Ph OLEDs based on the CHM-HENA-D route and other representative amorphous hosts. The CHM-HENA-D OLED has the lowest turn-on voltage and driving voltage at 1000 cd/m^2^ and simultaneously shows high EQE and excellent color purity in these OLEDs.

**Table 1.** Key performance parameters of DSA-Ph OLEDs based on CHN-HENA-D route and representative amorphous hosts. FWHM, full width at half maximum; MADN, 2-methyl9,10-di(2-napthyl)anthracene; MBA, 3,3'-dimethyl-9,9'-bianthracene; TBBA, 9,9'-bi(3,3'-tert-butyl)anthracene.

Hosts	*V*_on_/*V*_1000_/∆V* (V)	CE/PE/EQE_max_^†^ (cd A^−1^/lm W^−1^/%)	CE/PE/EQE^‡^ (cd A^−1^/lm W^−1^/%)	λ_max_^§^ (nm)	FWHM (nm)	CIE (*x*, *y*)	(CE_max_/CIE_*y*_)_max_^║^	Ref.
2FPPICz:PAC nanoaggregate	2.5/3.3/0.8	12.14/13.61/9.14	9.43/9.08 /7.10	464	50	(0.15, 0.17)	71.4	This work
DA-type 1,3,5,9-tetraarylpyrenes	2.6/3.7^¶^/1.1	12.95/13.56/6.84	11.3^¶^/6.1^¶^/5.5^¶^	468	60^¶^	(0.15, 0.28)	46.3	(*44*)
MADN	2.8/3.8/1.0	16.0^¶^/14.0^¶^/−	15.8/13.0/7.2	500^¶^	67^¶^	–	–	(*8*)
MBA	3.0/5.4^¶^/2.4^¶^	16.54/16.57/9.47	16.4^¶^/10.2^¶^/9.3^¶^	468	55	(0.15, 0.26)	63.6	(*55*)
TBBA	2.9/6.3^¶^/3.4^¶^	11.33/10.45/7.16	11.2¶/5.4^¶^/7.0^¶^	460	60	(0.15, 0.20)	56.7	(*55*)

*V_on_ and *V*_1000_ are operation voltage at the brightness of 1 and 1000 cd/m^2^, respectively, and ∆*V* is the difference between *V*_on_ and *V*_1000_.

†Maximum CE, PE, and EQE values.

‡CE/PE/EQE values of the devices at a luminance of 1000 cd/m^2^.

§Maximum emission wavelength.

║Blue index [ratio of the maximum current efficiency (CE_max_) to CIE*_y_*].

¶Estimated values based on figures in the corresponding literature.

### Photon output characteristic

**Fig. 5.**
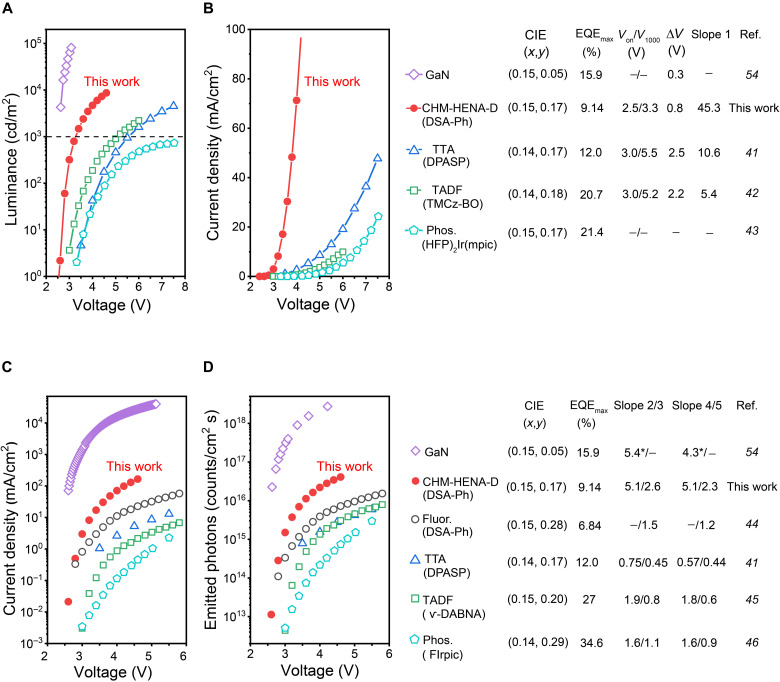
Comparisons of CHM-HENA-D OLED with A-OLEDs. (**A**) Comparison of voltage (*V*)–luminance and (**B**) voltage (*V*)–dependent current density (*J*), between the CHM-HENA-D OLED (2 wt % DSA-Ph) and reported blue OLEDs with similar color purity. An inorganic LED based on lnGaN/GaN is also plotted as a reference (*54*). ∆*V* = *V*_1000_(1000 cd/m^2^) − *V*_0_(1 cd/m^2^). (**C**) Comparison of voltage-dependent semi-log density. (**D**) Semi-log emitted photons (*N*), between the CHM-HENA-D OLED and reported OLEDs based on fluorescent, phosphorescent, and TADF materials, and lnGaN/GaN LED (*54*). *Instantaneous slope at the lowest luminance (4273 cd/m^2^) shown in (*54*). All reference data for comparison are extracted from the corresponding literature.

The C-OLEDs are capable of achieving much enhanced photoemission at the same driving voltage in OLEDs, owing to the high carrier mobility of crystalline thin films and the resultant rapid formation of excitons (*24*, *40*). Figure 5A shows the comparison of the brightness of the CHM-HENA-D OLED (2 wt % DSA-Ph) with reported typical blue OLEDs with high EQE, of which the CIE*_y_* coordinate values are close to but less than 0.20 (*41*–*43*). The CHM-HENA-D OLED has the lowest turn-on voltage and the fastest brightness climb as the driving voltage increases, that is, the smallest driving voltage at the same brightness after the devices are turned on. The brightness needs only an increment of 0.8 V switching from 1 to 1000 cd/m^2^ in the CHM-HENA-D OLED, which is the smallest value among all the OLEDs with similar color purity. There is no doubt that the rapid increase in brightness plays an important role in the practical application of OLEDs. The CHM-HENA-D OLED also has the fastest ramping of current density with increasing driving voltage (Fig. 5B), revealing that the CHM-HENA-D OLED has a much higher conductance, owing to the high carrier mobility of the crystalline thin film. Slope 1 is defined as the instantaneous slope of *J-V* curves at their corresponding luminance of approximately 1000 cd/m^2^, i.e., areal differential conductance of these OLEDs. Several electric parameters (e.g., input power, differential resistance, and Joule heat loss of series resistance) are acquired in table S3. At a luminance of approximately 1000 cd/m^2^, the CHM-HENA-D OLED has the lowest areal differential resistance (0.0221 kilohms·cm^2^) and ratio of series resistance Joule heat to input power (7.8%), demonstrating that the inevitable energy loss of operating OLEDs can be effectively decreased by using the CHM-HENA-D route. To further explore the photon output characteristics of CHM-HENA-D OLEDs, a voltage-dependent semi-log of current density is used to analyze the rate of conductance change at low operation voltages of OLEDs with various blue-emitting mechanism materials (Fig. 5C), including fluorescent (*44*), TTA (*41*), TADF (*45*), and phosphorescent (*46*). The areal differential conductance at 1000 cd/m^2^ and electric parameters of these OLEDs are also obtained in table S3. Slope 2 and Slope 3 represent the instantaneous slope of the log(*J*)-*V* curves at 10 cd/m^2^ and the average slope from 10 to 1000 cd/m^2^. The highest Slope 2 and Slope 3 values of the CHM-HENA-D OLED indicate that the high mobility of the crystalline thin film is beneficial to improving the conductance of the OLEDs and achieving fast turning-on at low driving voltages. To objectively evaluate the emission ability of OLEDs, the quantity of emitted photons per unit time per unit area (*N*) can be used as an evaluation standard to eliminate the impact of human visual function. *N* was calculated from the current density (*J*) and the EQE, that is, *N* = EQE×*J*/*e*, where *e* is the elementary charge. It is easy to understand that the large photon output originates from the high EQE and high conductance of OLEDs. Slope 4 and Slope 5 are defined as the instantaneous of log(*N*)-*V* curves at 10 cd/m^2^ and the average from 10 to 1000 cd/m^2^, respectively. As illustrated in Fig. 5D, the highest Slope 4 and Slope 5 values of the CHM-HENA-D OLED indicate that, owing to the high carrier mobility of crystalline thin films and the collection of hot excitons, the prominent conductance and sufficient EQE are deterministic for achieving the largest number of photons emitted under the same operation voltage compared to reported high-efficiency blue OLEDs. It is worth noting that the photons emitted by the CHM-HENA-D OLED (2 wt % DSA-Ph) are more than one order of magnitude larger than those based on blue A-OLEDs at 4 V. These results demonstrate that the C-OLEDs sensitized by PAC nanoaggregates produce a stronger light emission and faster ramping slope than all present high-efficiency OLEDs.

### Thermal stability of CHM-HENA-D thin film

The thermal stability of thin films is a key factor affecting the lifetime of devices (*47*, *48*). By repeating alternate growth of PAC nanoaggregates with 2 wt % DSA-Ph and 2FPPICz crystalline thin film, the 20-nm-thick CHM-HENA-D thin film was prepared on Si/SiO_2_/BP1T crystalline thin film (6 nm)/2FPPICz crystalline (5 nm) substrate, which is named as Thin Film MS1. We studied the time-dependent morphology evolution of Thin Film MS1 by AFM characterization in an atmospheric environment [25°C; humidity, 50 to 60% (relative humidity (RH); without encapsulation]. Figure S12 (A to F) shows the morphology evolution images of Thin Film MS1 with the time of 0 (fig. S12A), 20 (fig. S12B), 50 (fig. S12C), 100 (fig. S12D), 200 (fig. S12E), and 300 hours (fig. S12F). It can be observed that the top parts of PAC nanoaggregates are homogeneously exposed out of the thin film, and the CHM-HENA-D thin film has a molecularly smooth surface roughness (RMS is about 2 nm). After being placed in the atmosphere for 300 hours without encapsulation, the morphology of the CHM-HENA-D thin film remains unchanged. Furthermore, thermal degradation is one of the major barriers to the production of longer-lifetime OLEDs. Although many techniques are used to dissipate the heat energy associated with OLED (*47*), the good thermal stability of thin films at high temperatures is highly demanded for extending the OLED lifetime. Therefore, the CHM-HENA-D thin-film stability in a high-temperature environment was further studied. Figure S12 (G to K) shows the AFM images of CHM-HENA-D thin film placed in an oven at a temperature of 80°C and a pressure of 300 Pa, with the time of 10 (fig. S12G), 30 (fig. S12H), 60 (fig. S12I), 100 (fig. S12J), and 200 hours (fig. S12K). It can be observed that the morphology of the CHM-HENA-D thin film has not changed. Figure S12l shows the out-of-plane XRD patterns of Thin Film MS1 after being maintained for 200 and 300 hours at 25°C and for 100 and 200 hours at 80°C, and the patterns of the thin film have not changed compared to the initial status. These results reveal that the CHM-HENA-D thin film has superior thermal stability, which is beneficial to realizing long-lifetime OLED devices.

In addition, a reference sample 2FPPICz: PAC (20 wt %): DSA-Ph (2 wt %) (50-nm) amorphous thin film, was prepared, which is named as Thin Film MS2. Figure S13 (B to H) shows the morphology evolution of the reference amorphous thin film in an atmospheric environment (25°C; humidity, 50 to 60% RH; without encapsulation) with the time of 0 (fig. S13B), 5 (fig. S13C), 10 (fig. S13D), 20 (fig. S13E), 40 (fig. S13F), 80 (fig. S13G), and 120 hours (fig. S13H). The flat and uniform amorphous thin film can be observed from the AFM images at the initial status. However, the phenomenon of phase separation and “large aggregates” appears in the time of only 5 hours, and phase separation gets more evident as time increases (large aggregates is about several hundreds of nanometers when the thin film was placed for 120 hours). The morphology evolution of Thin Film MS2 amorphous thin film in an environment with high temperature of 80°C and pressure of 300 Pa was also studied, of which the notable phase separation occurs just within several hours (in fig. S14). To understand the thermal stability, a material system used in long-lifetime A-OLED was studied and compared. Amorphous MADN:DSA-Ph (3%) thin film (50 nm) (Thin Film MS3) was used as an EML in a long-lifetime OLED [*T*_1/2_ = 46,000 hours (*49*)], whose morphology evolution in an atmospheric environment (25°C; humidity, 50 to 60% RH; without encapsulation) with the time of 0 (B), 10 (C), 20 (D), 30 (E), 40 (F), 60 (G), and 120 hours (H) is demonstrated in fig. S15. In the beginning, the amorphous thin film is flat and uniform. However, organic molecules start to aggregate, and “pinholes” appear in the thin film as time increases. For the experiment at an 80°C environment (pressure, 300 Pa), fig. S16 (A to D) shows the morphology evolution of Thin Film MS3 with the time of 1 (fig. S16A), 3 (fig. S16B), 5 (fig. S16C), and 10 hours (fig. S16D). It can be observed that notable phase separation occurs in an amorphous thin film within several hours under an environment with a high temperature of 80°C and pressure of 300 Pa. In summary, compared to these amorphous thin films of 2FPPICz: PAC: DSA-Ph counterpart and typical materials MADN:DSA-Ph used in the long-lifetime A-OLED, the 2FPPICz CHM-HENA-D thin film exhibits promising properties on thermal stability, which provides a potentially powerful route to develop long-lifetime OLED devices.

## DISCUSSION

Organic micro/nanostructures (e.g., micro/nanocrystals, ribbons, disks, and aggregates) have abundant advanced optoelectronic properties compared to common amorphous thin-film forms (*50*). For instance, the high mobility of organic crystals can effectively promote the charge transport and recombination rate of carriers. OLEDs will be improved to a higher level if organic nanostructures can be introduced and their properties are fully used. However, all the time, it has been deeply believed that high-performance OLEDs based on discrete organic nanostructures can hardly be created. Here, we developed a novel type of high-efficiency C-OLED sensitized by hot exciton material PAC nanoaggregates. The nanoaggregates are located in the 2FPPICz CHM, without damaging the continuity of the crystalline thin film. The heterojunction effect between PAC nanoaggregates and 2FPPICz thin film leads to nearly barrier-free passage for electron injection into PAC nanoaggregates from the electron transport layer. In the meantime, the hole carriers can transport through crystalline layers to PAC nanoaggregates and recombine with electrons to form excitons in PAC. On the basis of the above results, the device can achieve a fast turn-on, and the current quickly rises as the driving voltage increases. Then, conventional blue fluorescent dyes were doped into PAC nanoaggregates to enhance radiation emission. The high efficiency of these C-OLEDs can be achieved by using PAC nanoaggregates to harvest excitons and transferring them to blue dopants via an energy transfer process. Meanwhile, owing to the barrier-free carrier transport and high mobility of crystalline thin films, these CHM-HENA-D OLEDs also have a low turn-on voltage, a high PE, an extremely low ratio of series resistance Joule heat to input power, and an overwhelming photon output capacity compared to all reported blue OLEDs. In addition, the CHM-HENA-D thin film shows superior thermal stability, which is beneficial to develop a long-lifetime device. This work demonstrates that the strategy of HENAs combined with crystalline thin films will be a promising route for next-generation OLEDs with ultimate performance.

## MATERIALS AND METHODS

### Materials selection

To design a high-efficiency CHM-HENA-D OLED, BP1T, 2FPPICz, and hot exciton materials PAC and BD1 were synthesized according to the previous reports (*15*, *51*–*53*). DSA-ph, DPAVBi, and TmPyPB were purchased from Luminescence Technology Corp. (Lumtec). All materials were purified twice by thermal gradient sublimation before use.

### Film and device fabrication

The preparation process of crystalline thin films and devices adopted similar methods introduced in the previous reports (*22*, *24*, *30*). Heavily doped n-type silicon wafers with a 300-nm thermal oxidized SiO_2_ layer [capacitance per unit area (C*_i_*) = 10 nF cm^−2^] were used as the substrates to investigate the crystalline thin film and nanoaggregate morphologies. The Si/SiO_2_ substrates were cleaned with acetone, alcohol, and deionized water in turn, then desiccated in high-purity nitrogen, and dried in a bake oven at 120°C. To measure the PLQY of DSA-Ph, a 120-nm-thick N,N'-bis-(1-naphthyl)-N,N'-diphenyl,1,1'-biphenyl-4,4'-diamine (NPB) amorphous thin film with 2 wt % DSA-Ph was prepared on quartz substrates, which were cleaned using the same method as Si/SiO_2_ substrates. ITO films (180 nm thick) with 10 ohms per square coated on glass substrates were used as anodes of OLEDs. The ITO substrates were first purged with detergent and then ultrasonicated in acetone, ethanol, and detergent water in sequence for 20 min. ITO substrates were dried in a bake oven at 120°C and then treated with oxygen plasma for 15 min. Then, PEDOT:PSS (Clevios P VP Al 4083) was spin-coated at 4000 rpm for 30 s to modify the anode and then dried in a bake oven at 120°C for 30 min. Last, the Si/SiO_2_, quartz, or ITO was transferred into a vacuum chamber at a pressure of under 10^−4^ Pa. The growth rates of BP1T and 2FFPICz crystalline thin films are approximately 4 to 10 Å/min at a substrate temperature of 102°C. The growth rates of PAC nanoaggregates and dopants are 10 Å/min and 0.05 to 0.6 Å/min, respectively, held at the same substrate temperature of 102°C, and the growth rates of NPB, TmPyPB, LiF, and Al are 1 to 2, 1 to 2, 0.05 to 0.08, and 10 to 15 Å/S, respectively, at the room temperature. The thicknesses of all organic thin films were monitored by a quartz-crystal microbalance. The effective emission area overlapping the area between the ITO and Al electrodes is 4.0 mm by 4.0 mm for each device.

### Film and device characterization

The hRISC, T_1_-T_2_, and T_1_-T_3_ processes of hot exciton PAC material have been verified in various solution (*15*). An SPI 3800/SPA 300 HV (Seiko Instruments Inc., Japan) atomic force microscope in tapping mode was used to acquire the morphologies of the thin films and nanoaggregates. The out-of-plane XRD patterns were acquired using a thin-film diffraction diffractometer (D8 Discover) with Cu Kα radiation (λ = 1.54056 Å) at 40 kV and 40 mA (Bruker, Germany). The in-plane XRD patterns were acquired using a Rigaku SmartLab XRD instrument with Cu Ka radiation (*l* = 1.54056 Å). GIWAXD measurement was performed at Shanghai Synchrotron Radiation Facility. The monochromatic wavelength of the light source was 1.24 Å, and the data were recorded by MAR 225 CCD. Angle-dependent PL intensities of the *p*-polarized beam were measured to fit the anisotropy factor Θ of DSA-Ph molecules. An Edinburgh Instrument FLS980 spectrometer was used to measure the PL spectra and transient PL decay curves. UV-visible absorption spectra were acquired using a Shimadzu UV-3600 spectrometer. The PLQY properties of PAC nanoaggregates, DSA-Ph in PAC nanoaggregates, and DSA-Ph in NPB thin film were measured with excitation wavelength of 380 nm by a Hamamatsu Photonics C9920-2. A Keithley source measurement system (Keithley 2400/2000) with a calibrated silicon photodiode was used to test the *J*-*V*-*L* characteristics of OLED devices under an ambient atmosphere. A monochromatic Al KR source provides photons with 21.22 eV for UPS and 1486.6 eV for XPS, respectively. The samples were taken out from the vacuum chamber, exposed to air, and then transferred to the UPS/XPS measuring chamber. The fs-TA spectra were recorded by an fs-TA system optical parametric amplifiers (OPA) (ORPHEUS-TWINS) equipped with a femtosecond pulse laser (PHAROS LC, 25 kHz) as the pump source and a broadband laser-driven light source (EQ99X LDLS) as the probe source. The pump wavelength was 380 nm and measured at room temperature. The EL spectra and CIE coordinates were recorded with a Spectra scan PR650 spectrophotometer. In addition, the EQE values were obtained on the basis of the current density, luminance, and EL spectra assuming a Lambertian distribution. All thin-film and device characterizations were performed under an ambient laboratory atmosphere at room temperature.

## Supplementary Materials

### This PDF file includes:

Figs. S1 to S16 Tables S1 to S3 References
