# Supplementary material for: High-efficiency blue-emission crystalline organic light-emitting diodes sensitized by “hot exciton” fluorescent nanoaggregates
Source: Sci Adv. 2022 Dec 14;8(50):eadd1757. doi: 10.1126/sciadv.add1757 (PMC9750145; doi:10.1126/sciadv.add1757)
Supplement: Supplementary file 1 — Figs. S1 to S16 Tables S1 to S3 References [file sciadv.add1757_sm.pdf]

Science Advances
AAAS

Supplementary Materials for

High-efficiency blue-emission crystalline organic light-emitting diodes sensitized by "hot exciton" fluorescent nanoaggregates

Jingjie Yang *et al.*

Corresponding author: Feng Zhu, zhufeng@ciac.ac.cn

Sci. Adv. **8**, eadd1757 (2022)
DOI: 10.1126/sciadv.add1757

This PDF file includes:

Figs. S1 to S16
Tables S1 to S3
References

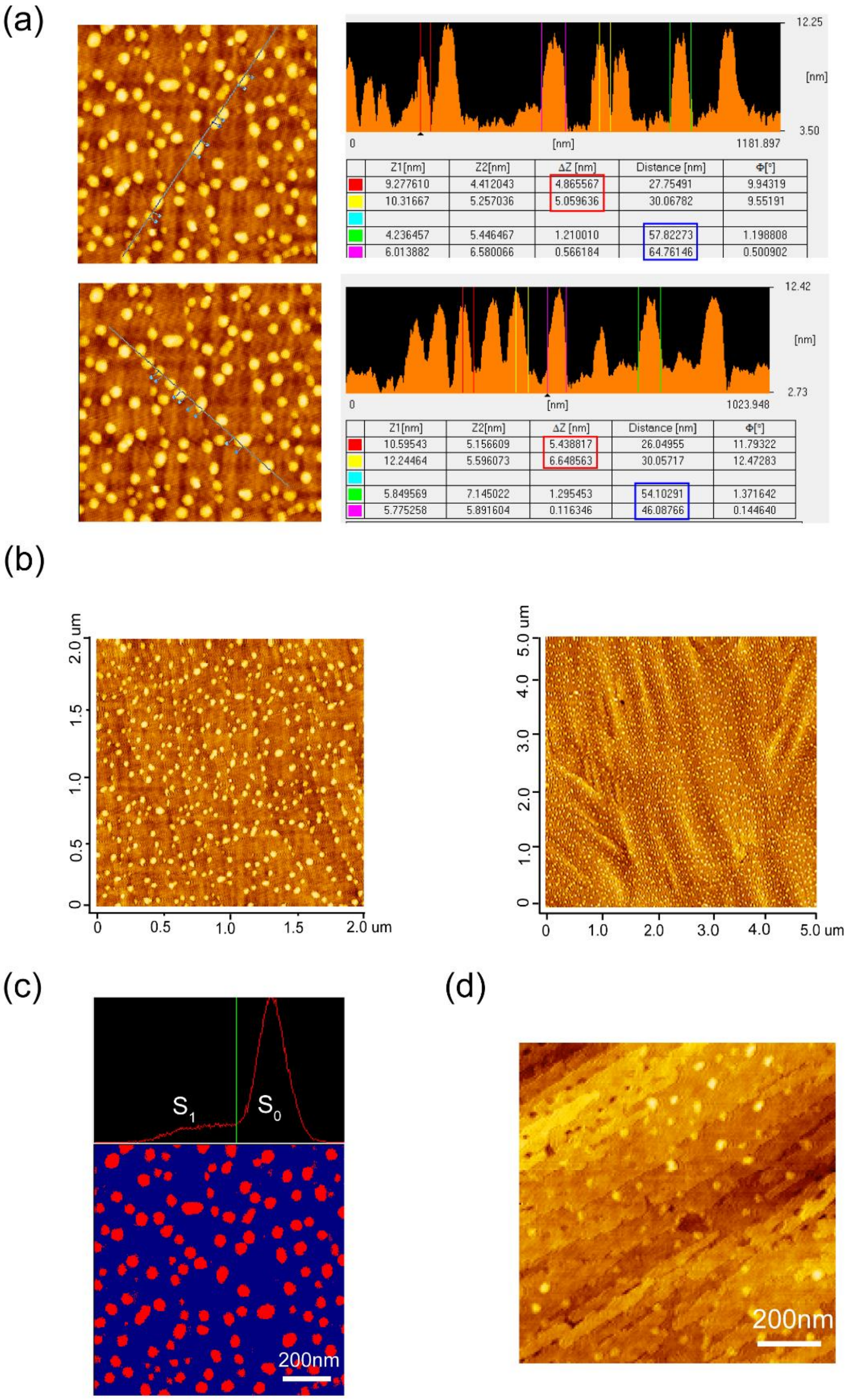

Fig. S1. **AFM images of nanoaggregates and crystalline thin films.** (a) AFM images of PAC nanoaggregates on 2FPPICz crystalline thin film and the crosssection profiles for estimating the height and diameter of PAC nanoaggregates. (b) AFM image of nanoaggregates with dimensions of 2 μm×2 μm and 5 μm×5 μm. (c) Processed AFM image for calculating the ratio of the vertical

projection area of PAC nanoaggreagtes on the 2FPPICz crystalline thin film. The integral area of the peak (red line) representing total area of 2FPPICz crystalline thin film S_0, the integral area of shoulder part (left to the green line) representing the area of PAC nanoaggregates on the 2FPPICz crystalline thin film S_1, the ratio is equal to S_1 / S_0 which is about 20%. (d) AFM image representing that 2FPPICz molecules are continuously deposited on top of 2FPPICz crystalline thin film with PAC nanoaggregates, forming a complete crystalline layer that covers the PAC nanoaggregates.

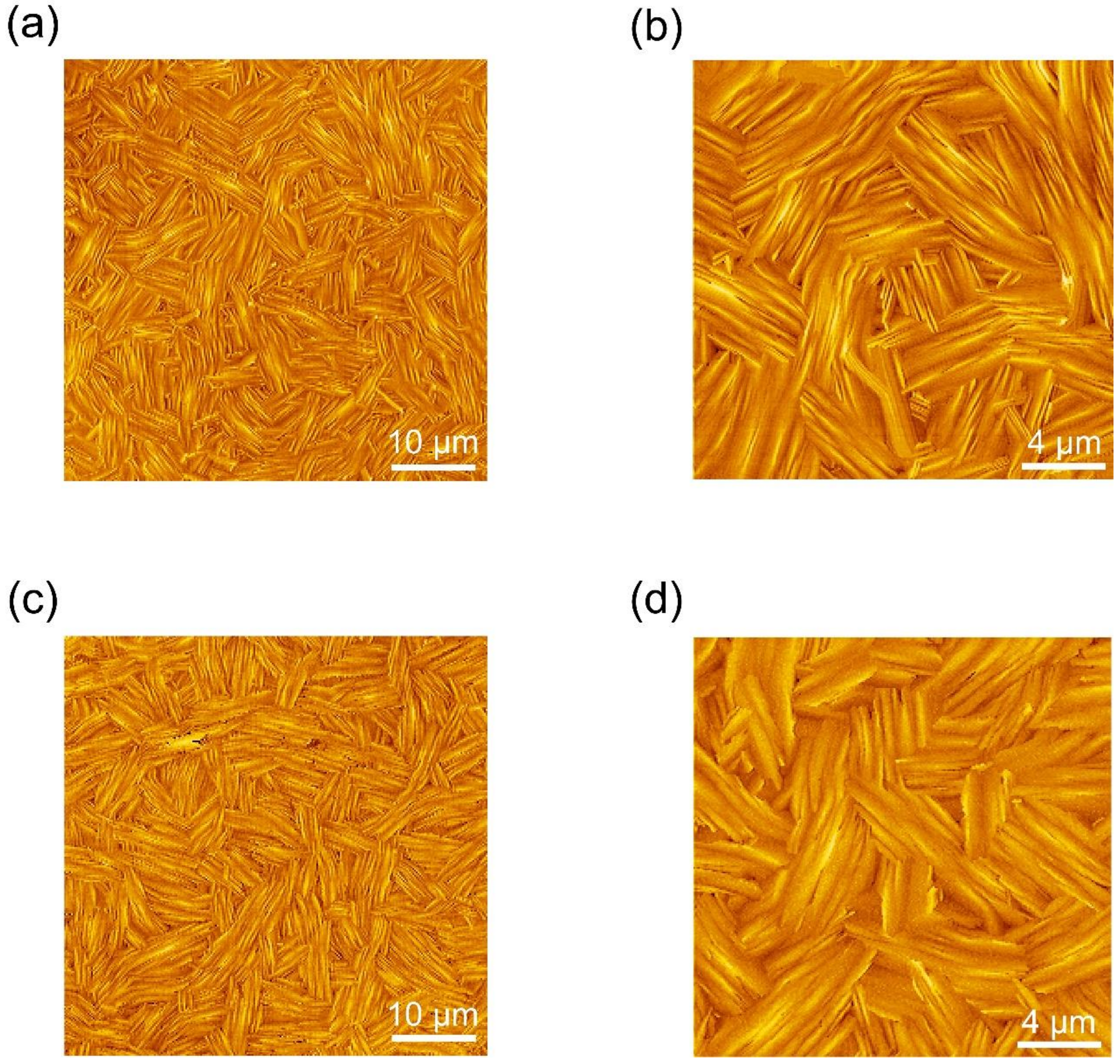

Fig. S2. AFM images of crystalline thin films. (a-b) AFM images of BP1T (6 nm) / 2FPPICz (25 nm) thin film with dimensions of 50 μm × 50 μm (a) and 20 μm × 20 μm (b). (c-d) AFM images of BP1T (6 nm) / 2FPPICz (5 nm) / CHM-HENA-D (20 nm) thin film with dimensions of 50 μm × 50 μm (c) and 20 μm × 20 μm (d).

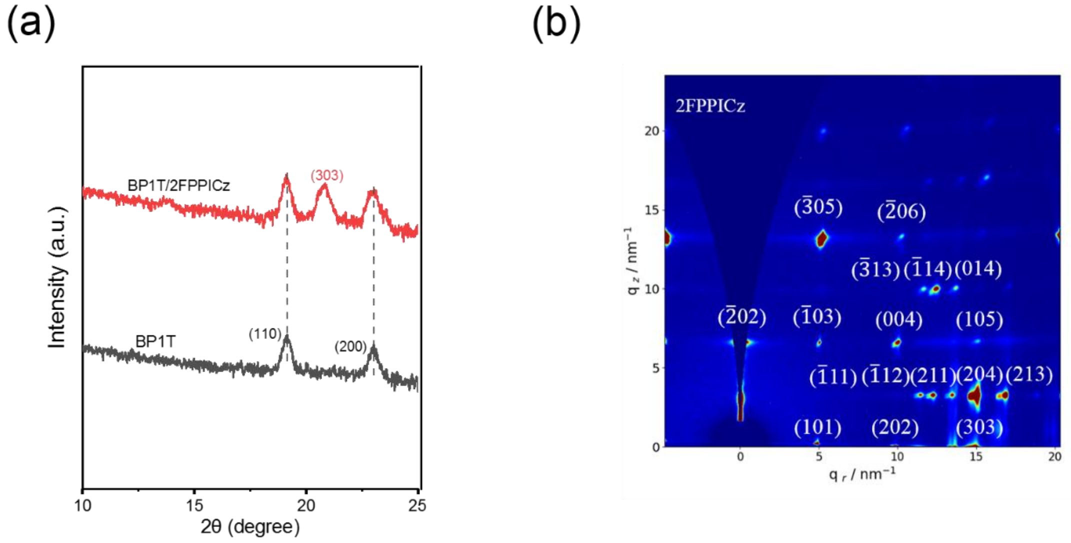

Fig. S3. XRD patterns of crystalline thin films. (a) In-plane XRD patterns of BP1T (6 nm) and BP1T (6 nm) / 2FPPICz (25 nm) crystalline thin films. (b) GIWAXD pattern of BP1T (6 nm) / 2FPPICz (60 nm) crystalline thin film.

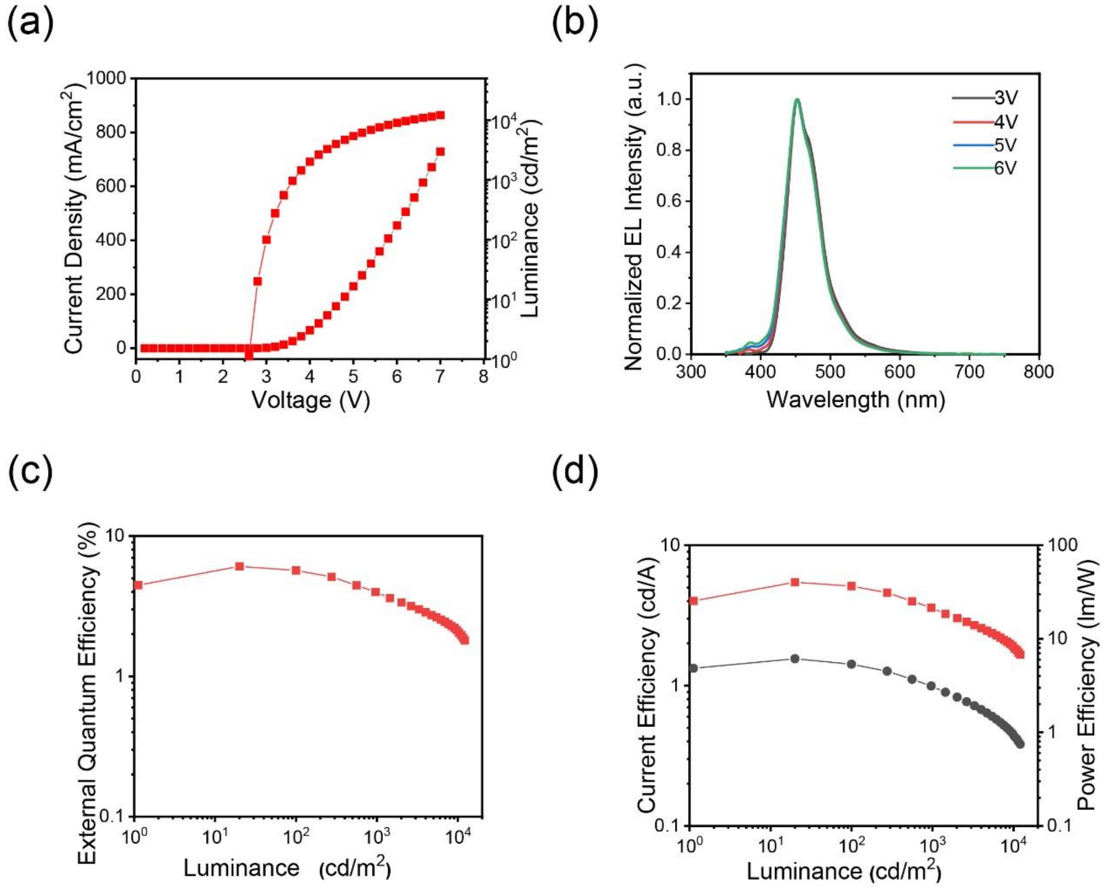

Fig. S4. Characterization of CHM-HENA OLED. (a) Current density and luminance versus voltage. (b) EL spectra of the device at different driving voltages. (c) EQE-luminance curve of the device. (d) Current efficiency (left) and Power efficiency (right) versus luminance.

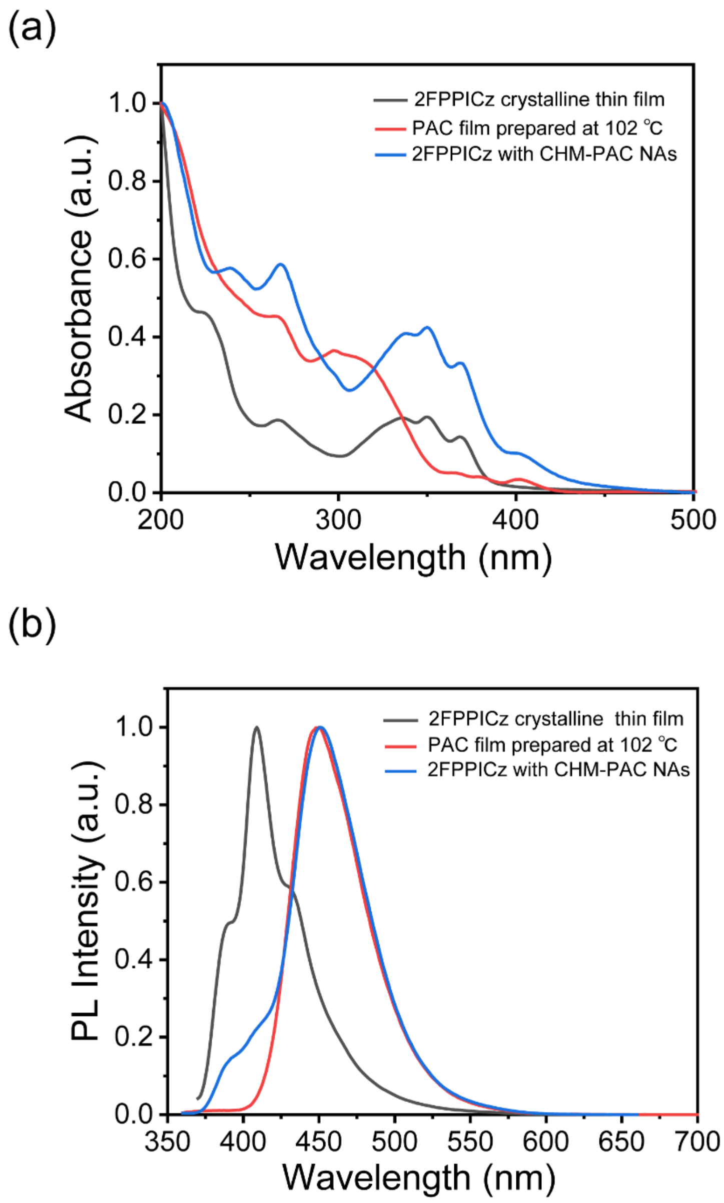

Fig. S5. Photophysical characterization. (a) Normalized ultraviolet-visible (UV-vis) absorption spectra of different films. (b) Photoluminescence (PL) spectra of different films. Note: 2FPPICz crystalline thin film was prepared on BP1T crystalline layer on quartz substrate at 102 °C. PAC film was prepared on quartz substrate at 102 °C. The excitation wavelength of PL spectrum is 350 nm.

(a)

(b)

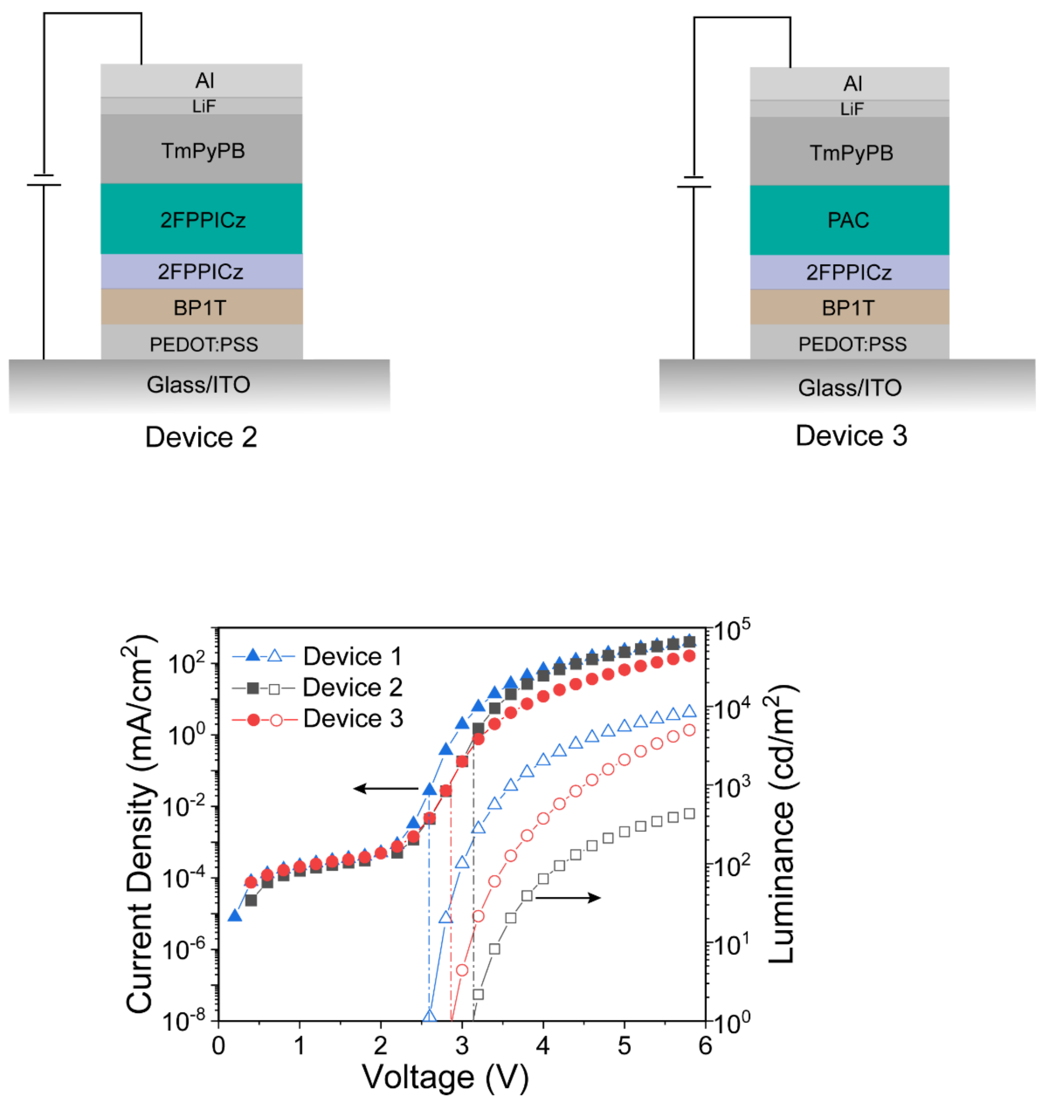

(c)

Fig. S6. Structure and characterization of devices. (a) Structure of Device 2. (b) Structure of Device 3. (c) Voltage-dependent current density and luminance of Device 1, Device 2, and Device 3.

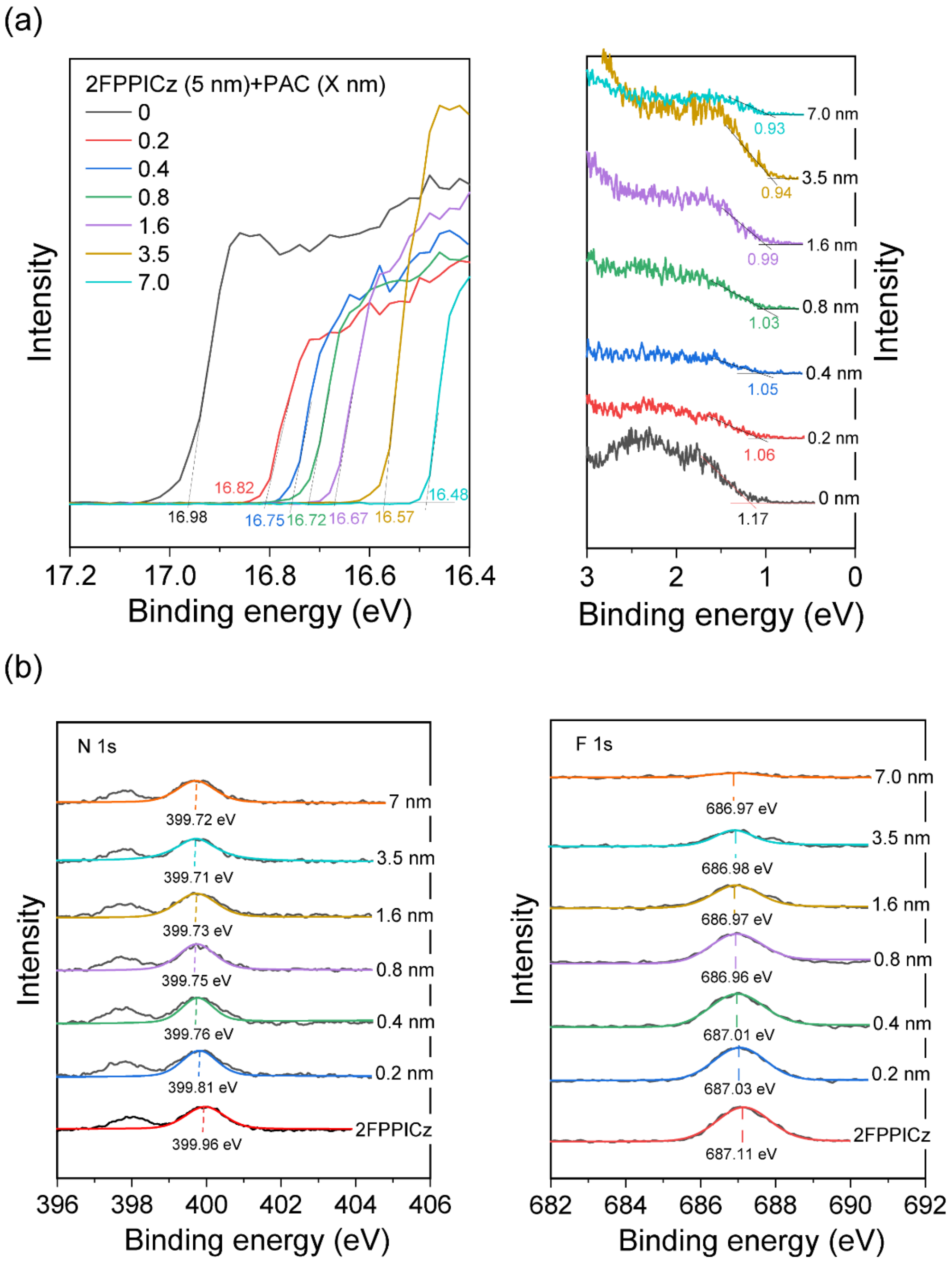

Fig. S7. UPS and XPS characterization. (a) Evolution of the secondary electron cutoff and the HOMO region of the UPS spectra as PAC is deposited on 2FPPICz crystalline film. (b) Evolution of the N 1s and F 1s core levels of XPS spectra as PAC is deposited on 2FPPICz crystalline film.

(a)

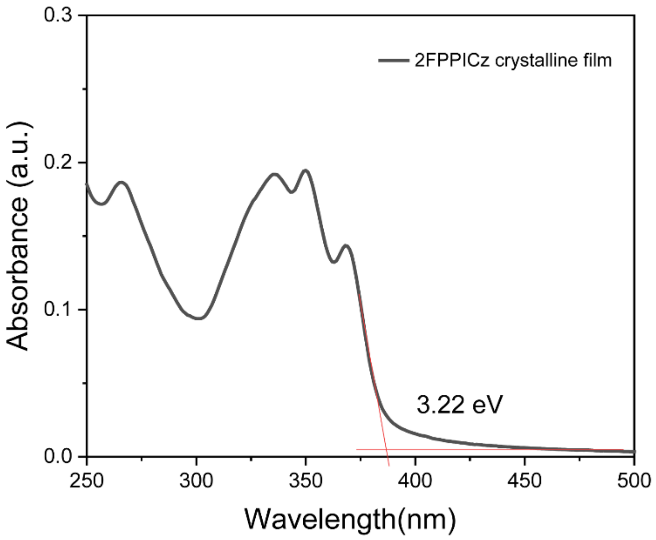

(b)

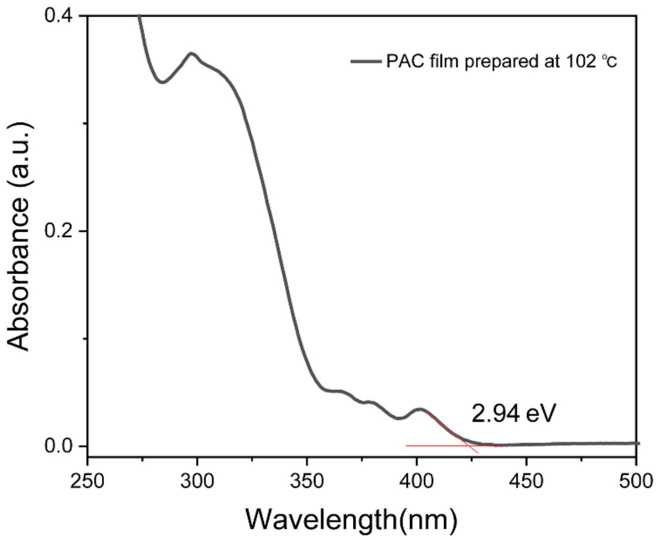

Fig. S8. Bandgap calculated from absorption spectra. (a) Bandgap of 2FPPICz crystalline film is calculated by the absorption band edge. (b) Bandgap of PAC is calculated by the absorption band edge.

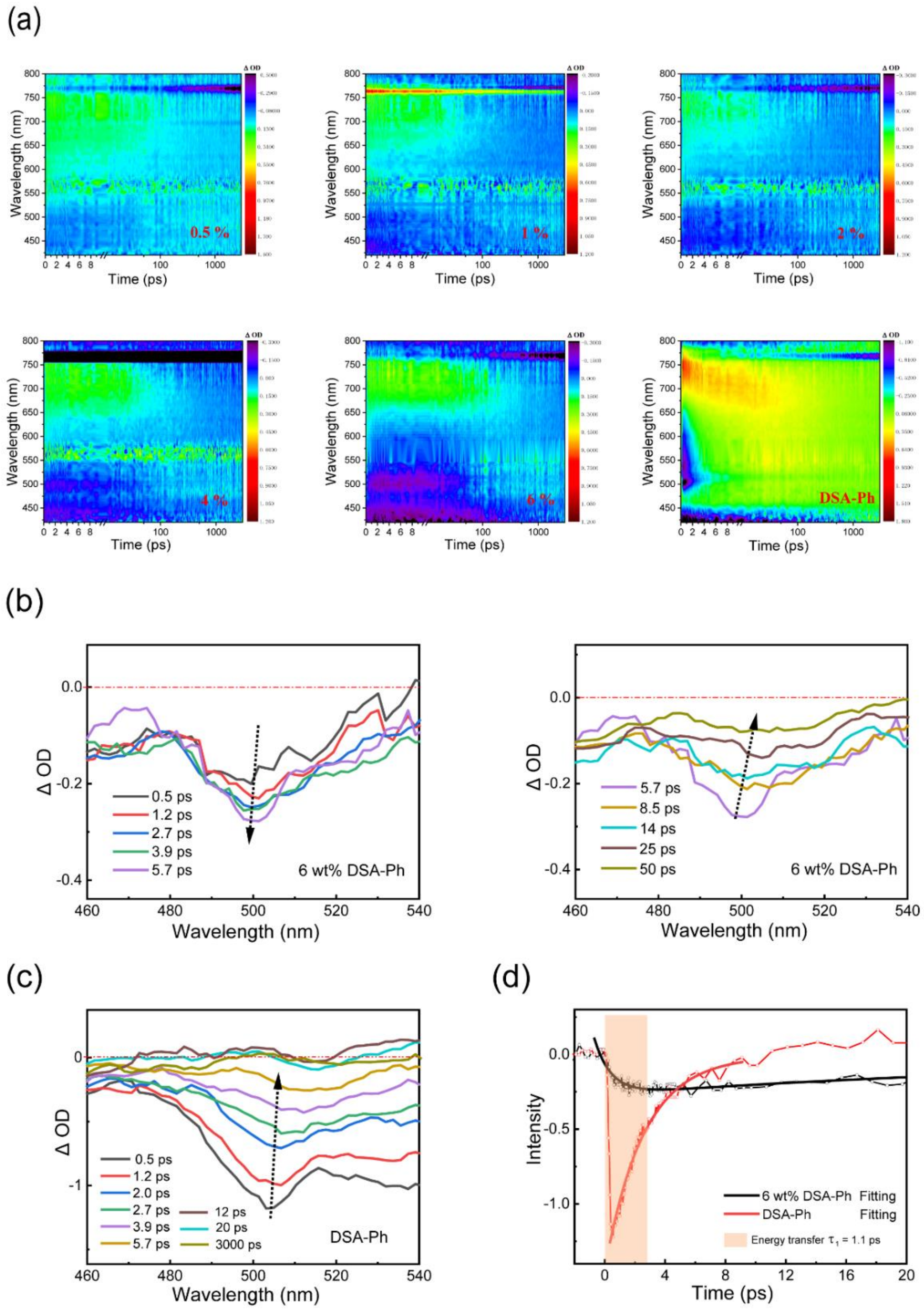

Fig. S9. Transient absorption characterization. (a) Transient absorption spectra of the PAC:DSA-Ph x wt% (x = 0.5, 1, 2, 4, 6) composite film and DSA-Ph film. (b) Transient absorption spectra recorded from PAC: DSA-Ph 6 wt% at different time delays. (c) Transient absorption spectra recorded from DSA-Ph film at different time delays. (d) Transient absorption kinetics for PAC: DSA-Ph 6 wt% and DSA-Ph film proded at 500 nm wavelengths as a function of delay time.

Transient absorption was performed to investigate the energy transfer process from PAC nanoaggregates to DAS-Ph and the energy transfer timescale. Several PAC films with different doped concentrations of DSA-Ph (0.5, 2, 4, 6 wt%) and DSA-Ph film were characterized by femtosecond transient absorption spectroscopy (fs-TA). As shown in Fig. S9a, the TA signal of these spectra at about 500 nm represents stimulated emission (SE) of DSA-Ph, and the SE signal intensity of doped films gradually increases with increasing the concentration, indicating that a sufficient energy transfer occurs from PAC nanaoaggregates to DSA-Ph as the concentration of DSA-Ph is increased. To further study the energy transfer dynamics, the spectra of the doped film with the concentration of 6 wt% and DSA-Ph pure film are selected to analyze the evolution of the SE signal. As shown in Fig. S9b,c, it is clearly observed that the SE signal intensity of DSA-Ph pure film gradually decreases to zero as time increases. For the SE signal intensity of the doped thin film with the concentration of 6 wt %, it is boosted first from 0.5 ps to 5.7 ps and then gradually decreases to zero. The difference of the SE signal evolution of DSA-Ph pure film and PAC doped thin film is attributed to the energy transfer from PAC to DSA-Ph and the timescale of energy transfer is about several picoseconds. As shown in Fig. S9d, the SE intensity of DSA-Ph pure film gradually decreases to zero as time increases, however, the evolution of SE intensity of the PAC doped thin film with the concentration of 6 wt% can be fitted by a multiexponential function. The fitting parameter τ_1 (1.1 ps) corresponds to the energy transfer timescale from PAC to DSA-Ph.

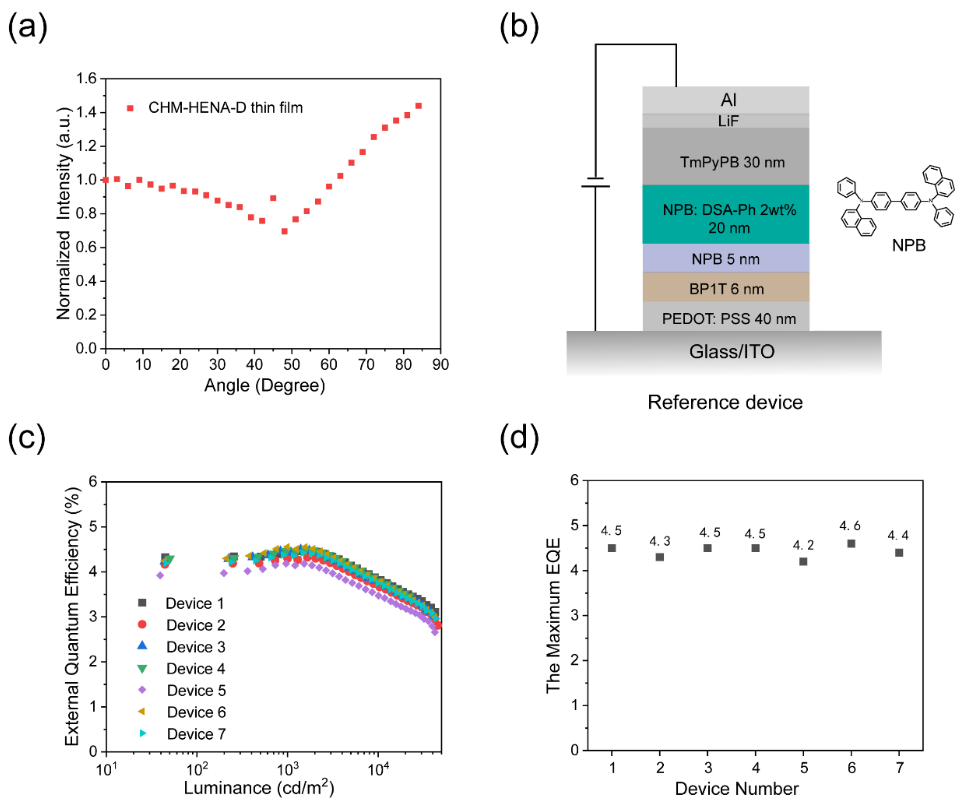

Fig. S10 . Characterization of CHM-HENA-D thin film and reference devices. (a) Horizontal dipole orientation characterizations of CHM-HENA-D thin film by angle-dependent p-polarized PL. (b) Structure of reference devices. (c) EQE-luminance curves of the reference devices. (d) Statistics of the maximum EQE of the reference devices.

The distinct orientation of the optical transition dipole moment vectors (TDMVs) of the emitting molecules is one of the important factors for η_{out} (*18*). The horizontally aligned TDMVs can greatly enhance the outcoupling of OLEDs (*37*) (*38*). Generally, in neat layers or homogeneous host-guest systems, the angle- and polarization-dependent luminescence(ADPL) curves of different TDMVs show different measured intensity (normalized to zero degrees emission) as a function of emission angle (*37*), which is, the measured intensity gradually decreases from zero to about 42°, and gradually increases to form a peak from about 42° to about 60°, then gradually decreases to zero until 90° because of total reflection of light. While horizontal dipoles couple most of their energy to directly emit, the vertical ones radiate mostly into substrate modes (*37*). Thus, the emission for angles higher than approximately 42° can be used to quantify

the relative ratio of horizontal to vertical dipoles. The weaker intensity represents more ratios of horizontal to vertical dipoles at about 42° (generally, if the organic molecules are randomly oriented, the measured intensity of isotropic dipoles is greater than 0.5) and the greater intensity represents more ratios of vertical to horizontal dipoles at about 60° (generally, if the organic molecules tend to be vertically oriented, the measured intensity of isotropic dipoles is greater than 1).

To determine the TDMVs of emitter DSA-Ph molecules in CHM-HENA-D thin film (BP1T (6 nm) / 2FPPICz (5 nm) / 2FPPICz CHM–HENA-D (30 nm)), ADPL measurement was performed. As shown in Fig. S10a, the measured minimum intensity is about 0.7 at 46°, indicating that the horizontal dipoles are not dominant. Besides, the measured intensity at about 60° is almost the same as that at zero degree, demonstrating that the DSA-Ph tends to be randomly oriented in CHM-HENA-D film. For the angle, more than 50°, the intensity increases as the angle increases instead of decreasing like a general case from 60° to about 90°, the reason for this abnormal phenomenon is assumed to be that the emission light is scattered by the surrounding nanoaggregates, so that the scattered light can be extracted at large angles. Therefore, the orientation of dopant molecules in CHM-HENA-D OLED is considered to be random in HENAs, and has no enhancement effect on the light outcoupling efficiency of the devices.

In addition, η_{out} also depends on the refractive indices and thicknesses of all individual layers (*37*). Therefore, several reference devices (Device 1 to Device 7), use a similar device structure to CHM-HENA-D OLED (Fig. S10b.), which was fabricated to clarify the η_{out}. The EQE-Luminance curves and the statistics of the maximum EQE are shown in Fig. S10c,d. The average maximum EQE of several reference devices is 4.4%. NPB and DSA-Ph as typical conventional fluorescent materials have been reported (*8*) (*56*). The maximum EUE of NPB and DSA-Ph is 25%, and Φ_{PL} of DSA-Ph in NPB thin films is 0.91. Therefore, the η_{out} factor of the reference device is calculated as 19.3%. The thicknesses of each layer in reference devices are the same as the ones in CHM-HENA-D OLED. Thus, CHM-HENA-D OLED has a similar η_{out} factor to that of the reference device, which is assumed to be approximately 20%.

Angle-dependent PL experiments were performed on a motorized rotational stage with a half cylinder lens made of fused silica. A continuous-wave UV laser was used as the excitation source and the incident angle of the laser was 45° to the detection plane. A linear polarizer was used to control the polarity of the emitted light. Angle-dependent p-polarized PL spectra of the film were detected by a fiber-guided spectrometer (USB 2000+, Ocean Optics Inc.) with an automately rotating stage. Emitting layer was deposited on a 0.2 mm-thick fused silica substrate. P-polarized light was used to analyze the orientation of the dipoles in the thin film.

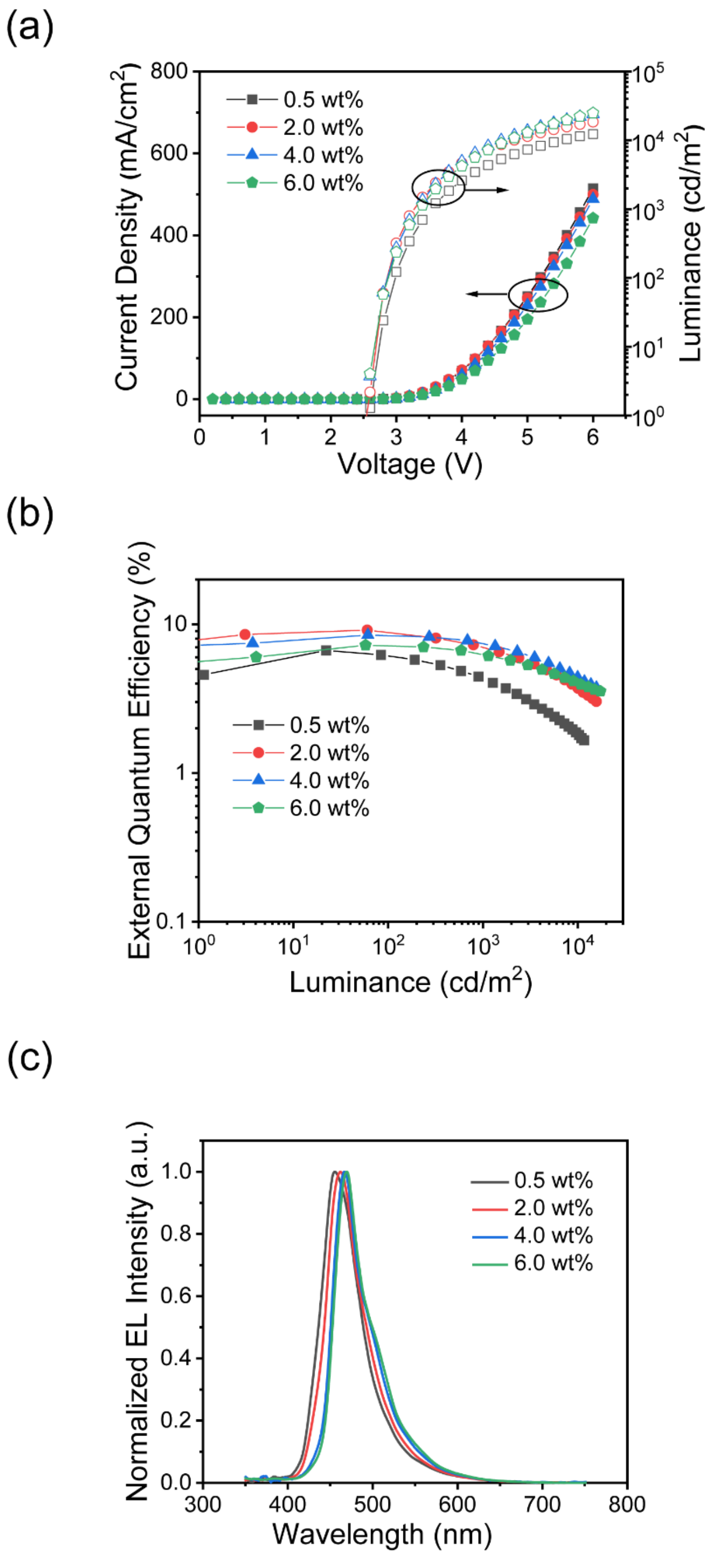

Fig. S11. Characterization of CHM-HENA-D with different concentrations of DSA-Ph. (a) Current density and luminance versus voltage. (b) EQE-luminance curves of the device. c) EL spectra of devices at 4 V.

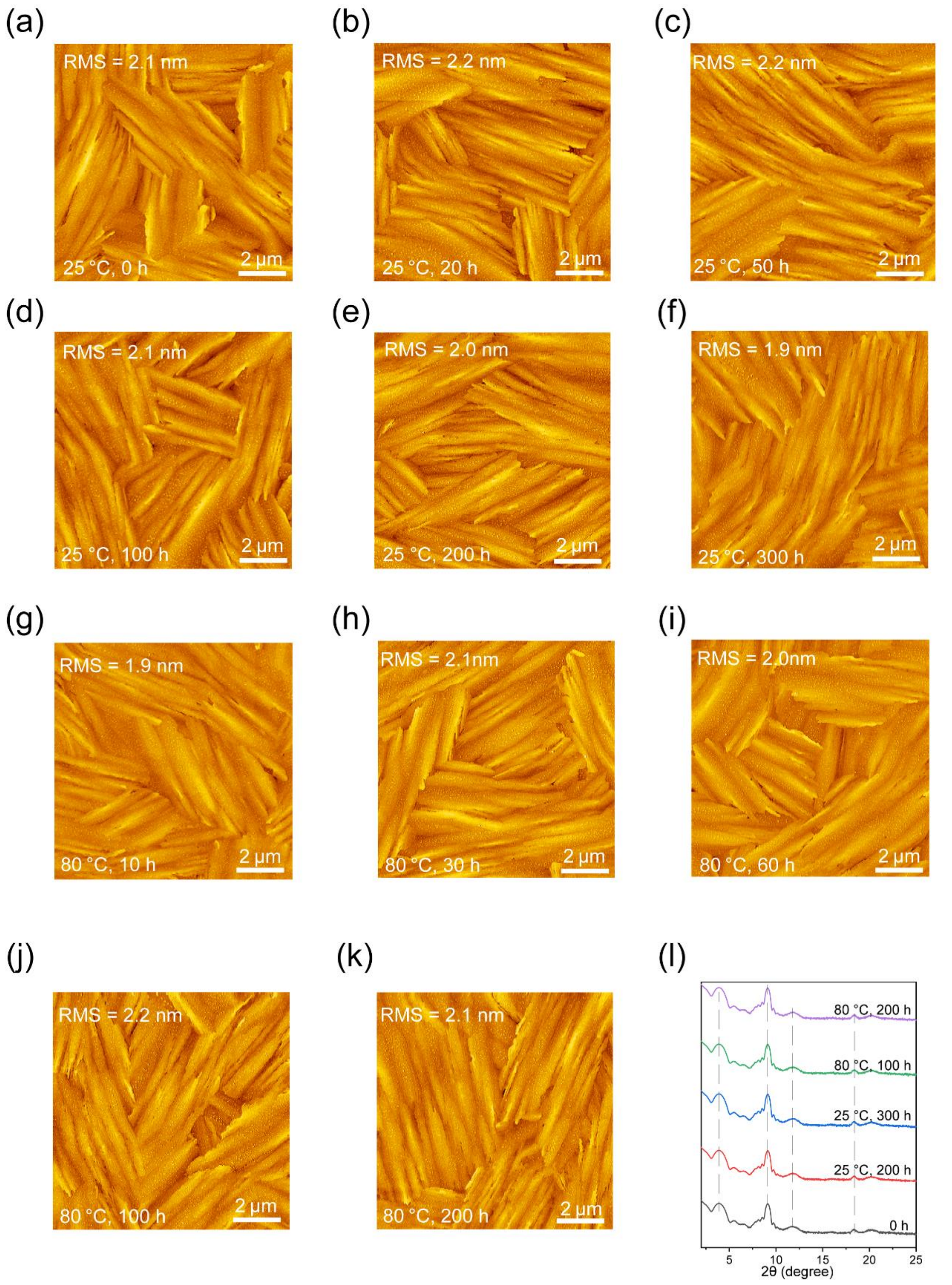

Fig. S12. Morphology evolution of Thin Film MS1. (a-f) Time-dependent AFM images of Thin Film MS1 thin film with the time of 0 h (a), 20 h (b), 50 h (c), 100 h (d), 200 h (e), 300 h (f) at 25 °C. (g-k) Time-dependent AFM images of Thin Film MS1 thin film with the time of 10 h (g), 30 h (h), 60 h (i), 100 (j), 200 h (k) at 80 °C. (l) Out-of-plane XRD patterns of Thin Film MS1.

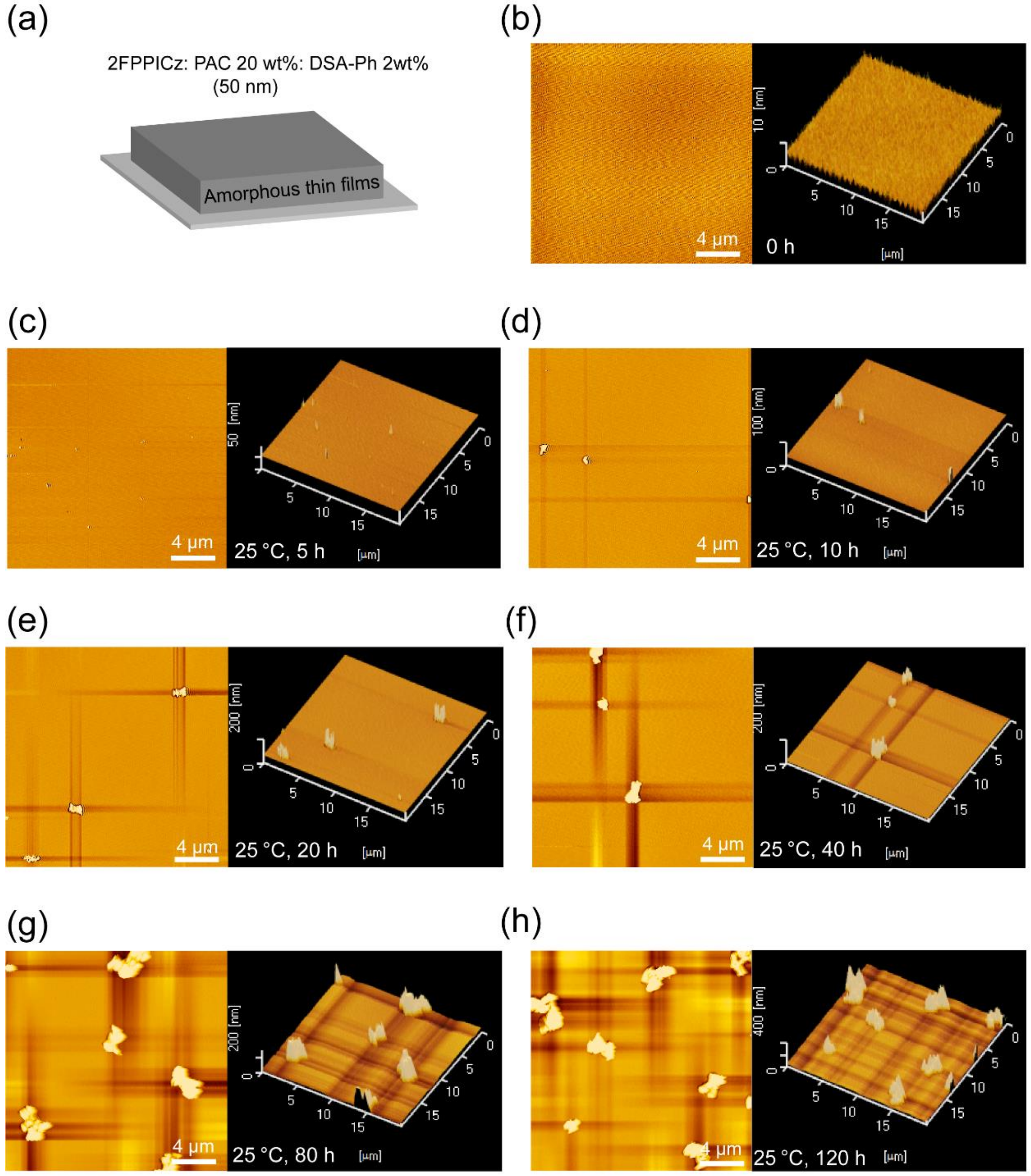

Fig. S13. Morphology evolution of Thin Film MS2. (a) Structure of Thin Film MS2. (b-h) Time-dependent AFM images of Thin Film MS2 with the time of 0 h (b), 5h (c), 10 h (d), 20 h (e), 40 h (f), 80 h (g), 120 h (h) at 25°C.

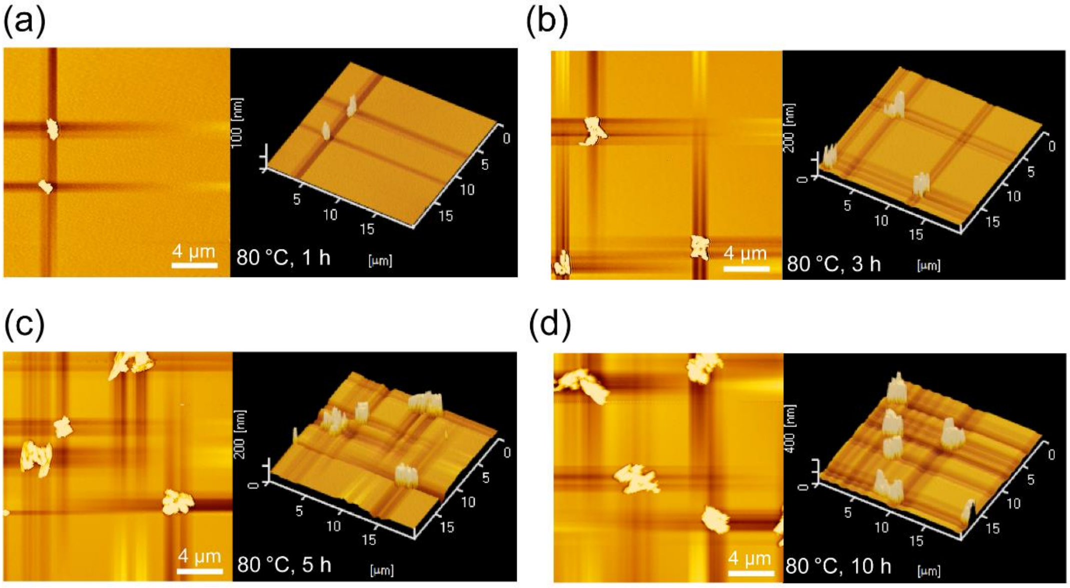

Fig. S14. Morphology evolution of Thin Film MS2. (a-d) Time-dependent AFM images of Thin Film MS2 with the time of 1h (a), 3h (b), 5 h (c), 10 h (d) at 80 °C.

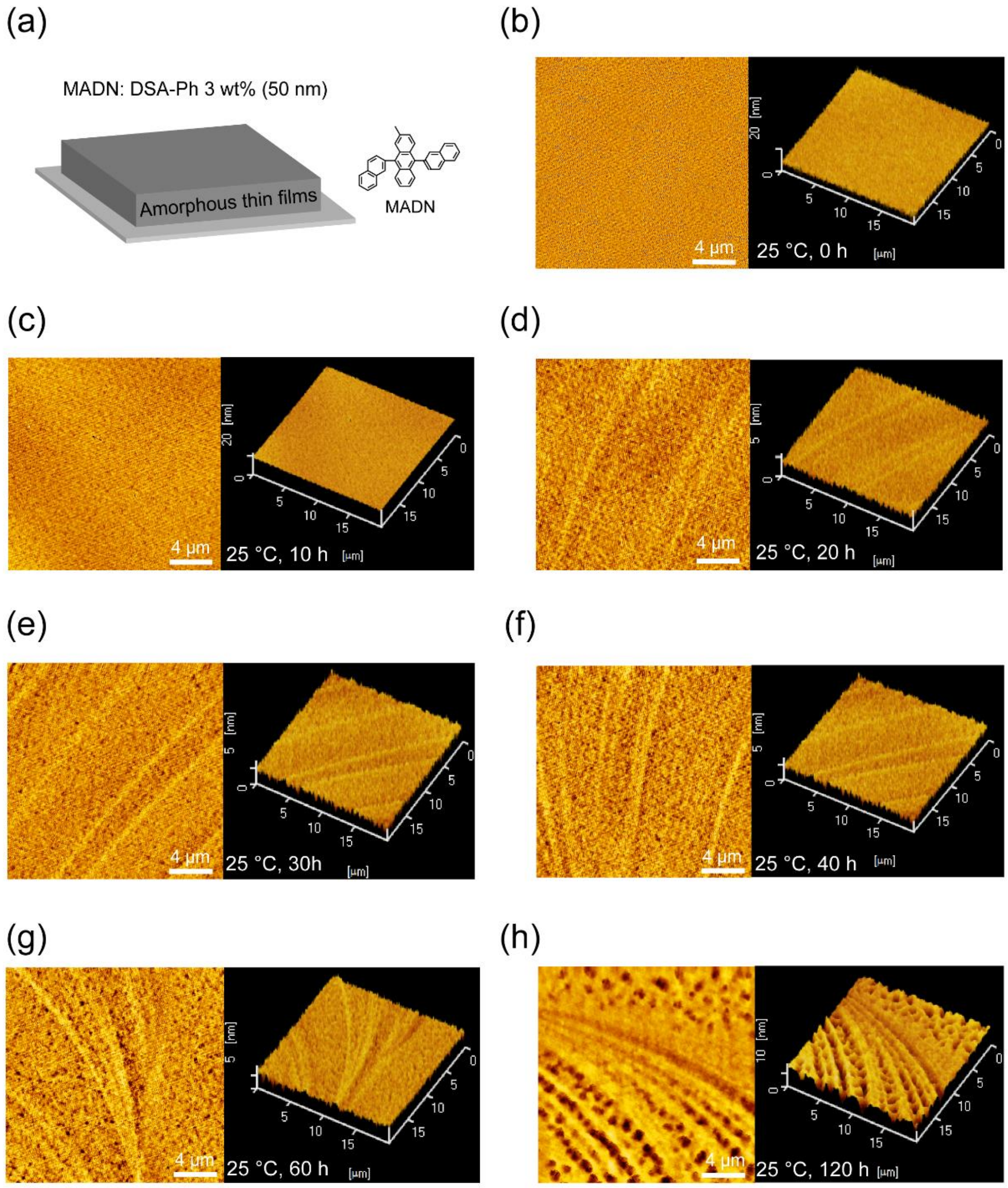

Fig. S15. Morphology evolution of Thin Film MS3. (a) Structure of Thin Film MS3. (b-h) Time-dependent AFM images of Thin Film MS3 with the time of 0 h (b), 10h (c), 20 h (d), 30 h (e), 40 h (f), 60 h (g), 120 h (h) at 25 °C.

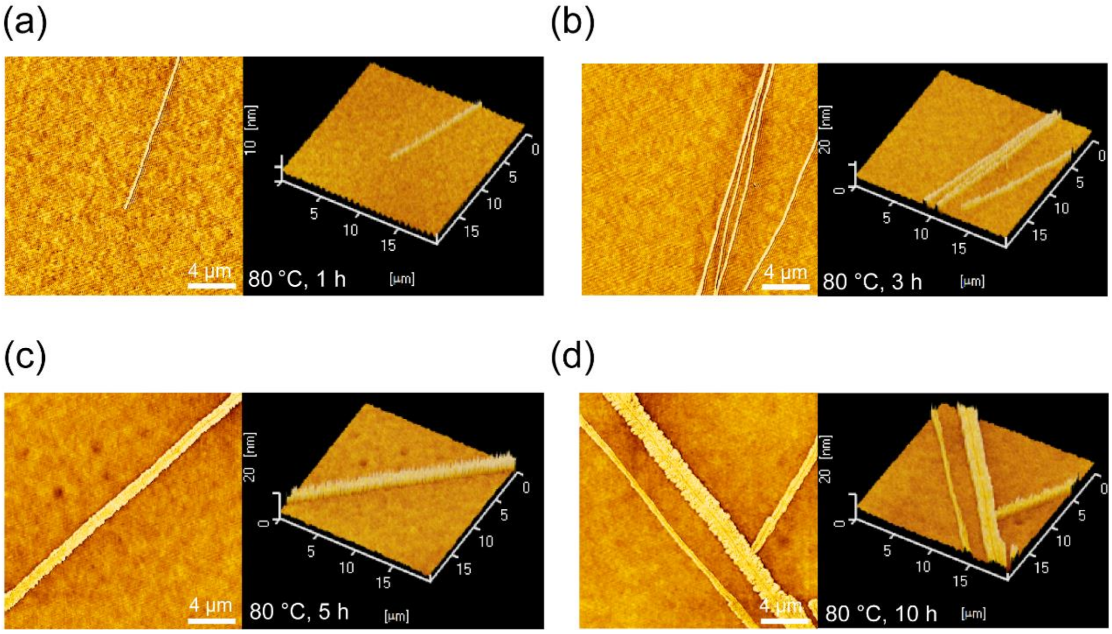

Fig. S16. Morphology evolution of Thin Film MS3. (a-d) Time-dependent AFM images of Thin Film MS3 with the time of 1h (a), 3h (b), 5 h (c), 10 h (d) at 80 °C.

Table S1. Summary of CHM-HENA-D OLEDs and amorphous thin-film sensitized blue fluorescent OLEDs with CIEy ≤0.2.

Emitters	$V_{on}/V_{1000}/\Delta V^{a}$ (V)	$CE/PE/EQE_{max}^{b}$ (cd A^{-1}/lm W^{-1}/%)	$CE/PE/EQE^{c}$ (cd A^{-1}/lm W^{-1}/%)	λ_{max}^{d} (nm)	CIE^{e} (X,Y)	Ref.
**CHM-HENA-D OLEDs**						
2FPPICz:PAC nanoaggregates:DSA-ph	2.5/3.3/0.8	12.14/13.61/9.14	9.43/9.08 /7.10	464	(0.15, 0.17)	This work
2FPPICz:PAC nanoaggregates:BD1	2.5/3.4/0.9	11.14/13.46/9.23	8.55/8.09/7.02	468	(0.14,0.19)	This work
2FPPICz:PAC nanoaggregates:DPAVBi	2.5/3.7/1.2	9.68/10.85/8.35	5.47/4.83/4.74	468	(0.16, 0.20)	This work
**Phosphorescent sensitized fluorescent emitters**						
mCBP:TSPO1:$(dfpysipy)_2Ir(mpic)$:TBPe	3.6/10.2*/6.6*	18.0/15.7/15.3	7.6*/2.36*/5.6	472*	(14, 0.19)	(*5*)
**TADF sensitizer fluorescentt emitters**						
DPEPO:DMAC-DPS:BPPyA	2.8*/5.1*/2.3	14.5/-/13.0	-/-/-	455*	(0.15, 0.16)	(*57*)
DBFPO:DMAC-DMT:BPPyA	3.0*/5.3*/2.3	18.3/-/19.0	-/-/-	457*	(0.14, 0.15)	(*57*)
**TTA sensitized fluorescent emitters**						
CzPA:Blue dopant	3.1/-/-	15.8/16.0/11.9	-/-/-	466	(0.14,0.17)	(*25*)
**Phosphorescent emitters**						
Ir(dbfmi)	2.56/4.74/2.18	28.6/35.9/18.6	9.4/6.3/6.2	454	(0.15,0.19)	(*58*)
$(fpmi)_2Ir(dmpypz)$	3.2/5.5*/2.3*	22.3/19.8/17.1	19.6/11.2/15.1	458	(0.13,0.16)	(*59*)
$(mmpi)_2Ir(dmpypz)$	3.2/5.5*/2.3*	21.9/19.1/15.4	19.3/11.0/13.6	464	(0.13,0.18)	(*59*)
**TADF emitters**						
CzVPN	4.4/11*/6.6*	18.0/12.3/8.7	-/-/3.2*	465	(0.15,0.18)	(*60*)
DCzTrz	3.5*/6.0*/2.5*	26.8/22.4/17.8	-/-/3.6*	468	(0.15,0.16)	(*61*)
DMT-DAc	3.0*/5.3*/2.3*	22.6/23.3/19.8	-/-/12.3*	451	(0.15,0.13)	(*62*)
DMAC-DPS	3.7/6.9*/3.2*	-/-/19.5	-/-/16.0	470	(0.16,0.20)	(*63*)
DTPDDA	3.0/5.7/2.7	35.6/30.4/22.3	17.9/9.8/10.6	468	(0.15,0.20)	(*64*)
34TCzTTrz	3.8*/9.8*/6.0*	19.1/15.0/10.3	-/-/<1	463	(0.16,0.20)	(*65*)
**"Hot exciton" materials**						
p-PTPAPI	3.2/5.7*/3.5*	3.7/3.5/3.7	3.3/1.8/3.2	450	(0.15,0.15)	(*66*)
TBPMCN	-/-/-	10.5/5.5/7.8	4.2/2.5/5.6	-	(0.16,0.16)	(*67*)
PAC	4.4/6.0*/1.6*	12.4/6.9/10.5	9.39*/4.8*/7.5	458	(0.15,0.13)	(*15*)
TPA-PPI	-/-/-	5.7/6.1/5.0	3.5/1.4/3.1	434	(0.15,0.11)	(*68*)
TPA-2PPI	2.8*/6.8*/4.0*	4.8/3.6/4.9	4.4/2.0/4.6	452	(0.15,0.11)	(*69*)
**TTA emitters**						
PIAnCN	3.0/5.4*/2.4*	13.16/-/9.44	-/-/9.44	470	(0.14,0.19)	(*12*)
TSTA	3.8/7.6*/3.8*	12.3/5.7/10.2	-/-/7.2*	454	(0.14,0.14)	(*70*)
3CzAnBzt	3.0/4.5*/1.5*	11.72/10.82/10.06	10.17/-/8.97	463	(0.14,0.14)	(*71*)
CBP:DPASP	3.0/5.5*/2.5*	18.5/16.5/12.0	-/-/7.2*	462	(0.14,0.17)	(*43*)
DMPPP:DPASP	2.6/5.5*/2.9*	13.0/8.9/10.7	-/-/10.5*	458	(0.14,0.14)	(*43*)

^{a} V_{on} and V_{1000} are operation voltages at the brightness of 1cd/m^2 and 1000cd/m^2, respectively, ΔV is the difference between V_{on} and V_{1000}. ^{b} Maximum current efficiency (CE), power efficiency (PE), and external quantum efficiency (EQE) values. ^{c} CE/PE/EQE values of the devices at luminance of 1000cd/m^2. ^{d} Maximum emission wavelength. ^{f} CIE:Commission Internationale de L'Eclairage. * Estimated values based on figures in the corresponding literatures.

Table S2. Photophysical parameters of PAC nanoaggregates: DSA-Ph *x* wt% (*x* = 0, 0.5, 2, 4, 6)

The EMLs	Φ_{PL} [a]	τ(ns) [b]	$K_r/10^8(S^{-1})$ [c]	$K_{nr}/10^8(S^{-1})$ [d]
PAC nanoaggregates	0.43	0.92	4.7	6.2
PAC nanoaggregates: DSA-Ph 0.5 wt%	0.60	1.09	5.5	3.7
PAC nanoaggregates: DSA-Ph 2 wt%	0.81	1.31	6.2	1.4
PAC nanoaggregates: DSA-Ph 4 wt%	0.84	1.78	4.7	1.0
PAC nanoaggregates: DSA-Ph 6 wt%	0.83	1.89	4.4	0.9

[a] PL quantum yield, [b] Lifetime of PL decay, [c] Rate constant of radiative transition,

[d] Rate constant of nonradiative transition

$$K_r = \frac{\Phi_{PL}}{\tau}$$

$$\tau = \frac{1}{K_r + K_{nr}}$$

$$K_{nr} = \frac{1}{\tau} - K_r$$

The rate constant of radiative transition (K_r) and rate constant of nonradiative transition (K_{nr}) can be calculated by the equations above.

Table S3. Summary of areal Joule heat loss of the CHM-HENA-D-OLED and other typical amorphous thin film blue-emission OLEDs at approximately 1000 cd/m^2

Emitters	Voltage (V)	Current density (mA/cm^2)	Input power (mW/cm^2)	Differential conductance (mS/cm^2)	Differential resistance (kΩ cm^2)	Joule heat loss (mW/cm^2)	Joule heat loss ratio (%)	CIE (x,y)
CHM-HENA-D (DSA-Ph) This work	3.3	11.6	38.3	45.3	0.0221	2.97	7.8	(0.15, 0.17)
TTA (DPASP) Ref. 43	5.5	13.0	71.5	10.6	0.094	15.9	22.2	(0.14, 0.17)
TADF (TMCz-Bo) Ref. 44	5.2	4.6	23.9	5.4	0.185	3.91	16.4	(0.14, 0.18)
Fluor. (DSA-Ph) Ref. 40	3.8	7.8	29.6	15.4	0.0649	3.95	13.3	(0.15, 0.28)
TADF (ν-DABNA) Ref. 46	5.6	5.7	31.9	5.0	0.200	6.50	20.4	(0.15, 0.20)
Phos. (FIrpic) Ref. 47	5.5	2.3	12.5	3.3	0.303	1.58	16.0	(0.14, 0.29)

REFERENCES AND NOTES

1. J. Kido, M. Kimura, K. Nagai, Multilayer white light-emitting organic electroluminescent device. *Science* **267**, 1332–1334 (1995).

2. S. Reineke, F. Lindner, G. Schwartz, N. Seidler, K. Walzer, B. Lussem, K. Leo, White organic light-emitting diodes with fluorescent tube efficiency. *Nature* **459**, 234–238 (2009).

3. M. A. Baldo, D. F. O'Brien, M. E. Thompson, S. R. Forrest, Excitonic singlet-triplet ratio in a semiconducting organic thin film. *Phys. Rev. B* **60**, 14422–14428 (1999).

4. Y. Im, S. Y. Byun, J. H. Kim, D. R. Lee, C. S. Oh, K. S. Yook, J. Y. Lee, Recent progress in high-efficiency blue-light-emitting materials for organic light-emitting diodes. *Adv. Funct. Mater.* **27**, 1603007 (2017).

5. H. G. Kim, H. Shin, Y. H. Ha, R. Kim, S. K. Kwon, Y. H. Kim, J. J. Kim, Triplet harvesting by a fluorescent emitter using a phosphorescent sensitizer for blue organic-light-emitting diodes. *ACS Appl. Mater. Interfaces* **11**, 26–30 (2019).

6. H. Nakanotani, T. Higuchi, T. Furukawa, K. Masui, K. Morimoto, M. Numata, H. Tanaka, Y. Sagara, T. Yasuda, C. Adachi, High-efficiency organic light-emitting diodes with fluorescent emitters. *Nat. Commun.* **5**, 4016 (2014).

7. S. Y. Byeon, D. R. Lee, K. S. Yook, J. Y. Lee, Recent progress of singlet-exciton-harvesting fluorescent organic light-emitting diodes by energy transfer processes. *Adv. Mater.* **31**, 1803714 (2019).

8. X.-F. Wei, W.-Y. Tan, J.-H. Zou, Q.-X. Guo, D.-Y. Gao, D.-G. Ma, J.-B. Peng, Y. Cao, X.-H. Zhu, High T_g small-molecule phenanthroline derivatives as a potential universal hole-blocking layer for high power-efficiency and stable organic light-emitting diodes. *J. Mater. Chem. C* **5**, 2329–2336 (2017).

9. L.-F. Chen, S.-T. Zhang, L. Hui, R.-F. Chen, J. Lu, Y. Kai, H.-H. Li, P. Lu, B. Yang, W. Huang, Breaking the efficiency limit of fluorescent OLEDs by hybridized local and charge-transfer host materials. *J. Phys. Chem. Lett.* **9**, 5240–5245 (2018).

10. S. H. Han, J. Y. Lee, Spatial separation of sensitizer and fluorescent emitter for high quantum efficiency in hyperfluorescent organic light-emitting diodes. *J. Mater. Chem. C* **6**, 1504–1508 (2018).

11. D. Y. Kondakov, Triplet–triplet annihilation in highly efficient fluorescent organic light-emitting diodes: Current state and future outlook. *Philos Trans A Math Phys Eng Sci* **373**, 20140321 (2015).

12. X.-Y. Tang, Q. Bai, T. Shan, J.-Y. Li, Y. Gao, F.-T. Liu, H. Liu, Q.-M. Peng, B. Yang, F. Li, P. Lu, Efficient nondoped blue fluorescent organic light-emitting diodes (OLEDs) with a high external quantum efficiency of 9.4% @ 1000 cd m^{-2} based on phenanthroimidazole−anthracene derivative. *Adv. Funct. Mater.* **28**, 1705813 (2018).

13. X. F. Qiao, D.-G. Ma, Nonlinear optoelectronic processes in organic optoelectronic devices: Triplet-triplet annihilation and singlet fission. *Mater. Sci. Eng. R Rep.* **139**, 100519 (2020).

14. W.-J. Li, Y.-Y. Pan, R. Xiao, Q.-M. Peng, S.-T. Zhang, D.-G. Ma, F. Li, F.-Z. Shen, Y.-H. Wang, B. Yang, Y.-G. Ma, Employing ~100% excitons in OLEDs by utilizing a fluorescent molecule with hybridized local and charge-transfer excited state. *Adv. Funct. Mater.* **24**, 1609–1614 (2014).

15. Y.-W. Xu, X.-M. Liang, X.-Z. Zhou, P.-S. Yuan, J.-D. Zhou, C. Wang, B.-B. Li, D.-H. Hu, X.-F. Qiao, X.-F. Jiang, L.-L. Liu, S.-J. Su, D.-G. Ma, Y.-G. Ma, Highly efficient blue fluorescent OLEDs based on upper level triplet-singlet intersystem crossing. *Adv. Mater.* **31**, 1807388 (2019).

16. H. Zhang, J.-N. Xue, C.-L. Li, S.-T. Zhang, B. Yang, Y. Liu, Y. Wang, Novel deep-blue hybridized local and charge-transfer host emitter for high-quality fluorescence/phosphor hybrid quasi-white organic light-emitting diode. *Adv. Funct. Mater.* **31**, 2100704 (2021).

17. V. Coropceanu, J. Cornil, D. A. da Silva Filho, Y. Olivier, R. Silbey, J.-L. Brédas, Charge transport in organic semiconductors. *Chem. Rev.* **107**, 926–952 (2007).

18. K. H. Kim, J. L. Liao, S. W. Lee, B. Sim, C. K. Moon, G. H. Lee, H. J. Kim, Y. Chi, J. J. Kim, Crystal organic light-emitting diodes with perfectly oriented non-doped Pt-based emitting layer. *Adv. Mater.* **28**, 2526–2532 (2016).

19. H. Nakanotani, C. Adachi, Organic light-emitting diodes containing multilayers of organic single crystals. *Appl. Phys. Lett.* **96**, 053301 (2010).

20. R. Ding, J. Feng, X.-L. Zhang, W. Zhou, H.-H. Fang, Y.-F. Liu, Q.-D. Chen, H.-Y. Wang, H.-B. Sun, Fabrication and characterization of organic single crystal-based light-emitting devices with improved contact between the metallic electrodes and crystal. *Adv. Funct. Mater.* **24**, 7085–7092 (2014).

21. J. Liu, H. Zhang, H. Dong, L. Meng, L. Jiang, L. Jiang, Y. Wang, J. Yu, Y. Sun, W. Hu, A. J. Heeger, High mobility emissive organic semiconductor. *Nat. Commun.* **6**, 10032 (2015).

22. X. X. Yang, X. Feng, J. H. Xin, P. L. Zhang, H. B. Wang, D. H. Yan, Highly efficient crystalline organic light-emitting diodes. *J. Mater. Chem. C* **6**, 8879–8884 (2018).

23. Y. Wan, J. Deng, W. Wu, J. Zhou, Q. Niu, H. Li, H. Yu, C. Gu, Y. Ma, Efficient organic light-emitting transistors based on high-quality ambipolar single crystals. *ACS Appl. Mater. Interfaces* **12**, 43976–43983 (2020).

24. J. H. Xin, P. F. Sun, F. Zhu, Y. Wang, D. H. Yan, Doped crystalline thin-film deep-blue organic light-emitting diodes. *J. Mater. Chem. C.* **9**, 2236–2242 (2021).

25. T. Suzuki, Y. Nonaka, T. Watabe, H. Nakashima, S. Seo, S. Shitagaki, S. Yamazaki, Highly efficient long-life blue fluorescent organic light-emitting diode exhibiting triplet–triplet annihilation effects enhanced by a novel hole-transporting material. *Jpn. J. of Appl. Phys.* **53**, 052102 (2014).

26. H. B. Wang, F. Zhu, J. L. Yang, Y. H. Geng, D. H. Yan, Weak epitaxy growth affording high-mobility thin films of disk-like organic semiconductors. *Adv. Mater.* **19**, 2168–2171 (2007).

27. J. L. Yang, D. H. Yan, Weak epitaxy growth of organic semiconductor thin films. *Chem. Soc. Rev.* **38**, 2634–2645 (2009).

28. L. S. Hung, C. H. Chen, Recent progress of molecular organic electroluminescent materials and devices. *Mater. Sci. Eng. R Rep.* **39**, 143–222 (2002).

29. D. H. Huh, G. W. Kim, G. H. Kim, C. Kulshreshtha, J. H. Kwon, High hole mobility hole transport material for organic light-emitting devices. *Synth. Met.* **180**, 79–84 (2013).

30. L. Liu, C. L. Li, Z. Q. Li, P. F. Sun, F. Zhu, Y. Wang, D. H. Yan, Highly oriented crystalline thin film with high electroluminescence performance fabricated by weak epitaxy growth. *Org. Electron.* **84**, 105806 (2020).

31. D. Liu, F. Zhu, D. Yan, Crystalline organic thin films for crystalline OLEDs (I): Orientation of phenanthroimidazole derivatives. *J. Mater. Chem. C* **10**, 2663–2670 (2022).

32. K. Walzer, B. Maennig, M. Pfeiffer, K. Leo, Highly efficient organic devices based on electrically doped transport layers. *Chem. Rev.* **107**, 1233–1271 (2007).

33. F. Zhu, M. Grobosch, U. Treske, M. Knupfer, L. Huang, S. Ji, D. Yan, Interfacial energy level bending in a crystalline p/p-type organic heterostructure. *Appl. Phys. Lett.* **98**, 203303 (2011).

34. I. Gutierrez Lezama, M. Nakano, N. A. Minder, Z. Chen, F. V. Di Girolamo, A. Facchetti, A. F. Morpurgo, Single-crystal organic charge-transfer interfaces probed using Schottky-gated heterostructures. *Nat. Mater.* **11**, 788–794 (2012).

35. M. Schwarze, W. Tress, B. Beyer, F. Gao, R. Scholz, C. Poelking, K. Ortstein, A. A. Gunther, D. Kasemann, D. Andrienko, K. Leo, Band structure engineering in organic semiconductors. *Science* **352**, 1446–1449 (2016).

36. X. Yin, G. Xie, T. Zhou, Y. Xiang, K. Wu, J. Qin, C. Yang, Simple pyridine hydrochlorides as bifunctional electron injection and transport materials for high-performance all-solution-processed organic light emitting diodes. *J. Mater. Chem. C* **4**, 6224–6229 (2016).

37. T. D. Schmidt, T. Lampe, D. Sylvinson M. R., P. I. Djurovich, M. E. Thompson, W. Brütting, Emitter orientation as a key parameter in organic light-emitting diodes. *Phys. Rev. Appl.* **8**, 037701 (2017).

38. K. H. Kim, J. J. Kim, Origin and control of orientation of phosphorescent and TADF dyes for high-efficiency OLEDs. *Adv. Mater.* **30**, 1705600 (2018).

39. D. Zhang, X. Song, M. Cai, L. Duan, Blocking energy-loss pathways for ideal fluorescent organic light-emitting diodes with thermally activated delayed fluorescent sensitizers. *Adv. Mater.* **30**, 1705250 (2018).

40. P. Martin, C. E. Swenberg, *Electronic Processes in Organic Crystals* (Oxford Univ. Press, 1982).

41. Y. H. Chen, C. C. Lin, M. J. Huang, K. Hung, Y. C. Wu, W. C. Lin, R. W. Chen-Cheng, H. W. Lin, C. H. Cheng, Superior upconversion fluorescence dopants for highly efficient deep-blue electroluminescent devices. *Chem. Sci.* **7**, 4044–4051 (2016).

42. J. U. Kim, I. S. Park, C. Y. Chan, M. Tanaka, Y. Tsuchiya, H. Nakanotani, C. Adachi, Nanosecond-time-scale delayed fluorescence molecule for deep-blue OLEDs with small efficiency rolloff. *Nat. Commun.* **11**, 1765 (2020).

43. J. B. Kim, S. H. Han, K. Yang, S. K. Kwon, J. J. Kim, Y. H. Kim, Highly efficient deep-blue phosphorescence from heptafluoropropyl-substituted iridium complexes. *Chem. Commun.* **51**, 58–61 (2015).

44. H. J. Ran, Z. Zhao, X. W. Duan, F. L. Xie, R. J. Han, H. M. Sun, J. Y. Hu, Blue-emitting butterfly-shaped donor-acceptor-type 1,3,5,9-tetraarylpyrenes: Easily available, low-cost conventional fluorophores for high-performance near ultraviolet electroluminescence with $CIE_y < 0.05$. *J. Mater. Chem. C.* **9**, 260–269 (2021).

45. C.-Y. Chan, M. Tanaka, Y.-T. Lee, Y.-W. Wong, H. Nakanotani, T. Hatakeyama, C. Adachi, Stable pure-blue hyperfluorescence organic light-emitting diodes with high-efficiency and narrow emission. *Nat. Photonics* **15**, 245–245 (2021).

46. D. L. Li, J. Y. Li, D. Liu, W. Li, C. L. Ko, W. Y. Hung, C. H. Duan, Highly efficient simple-structure sky-blue organic light-emitting diode using a bicarbazole/cyanopyridine bipolar host. *ACS Appl. Mater. Interfaces* **13**, 13459–13469 (2021).

47. S. Scholz, D. Kondakov, B. Lussem, K. Leo, Degradation mechanisms and reactions in organic light-emitting devices. *Chem. Rev.* **115**, 8449–8503 (2015).

48. S. Sudheendran Swayamprabha, D. K. Dubey, Shahnawaz, R. A. K. Yadav, M. R. Nagar, A. Sharma, F. C. Tung, J. H. Jou, Approaches for long lifetime organic light emitting diodes. *Adv. Sci.* **8**, 2002254 (2021).

49. M.-T. Lee, H.-H. Chen, C.-H. Liao, C.-H. Tsai, C. H. Chen, Stable styrylamine-doped blue organic electroluminescent device based on 2-methyl-9,10-di(2-naphthyl)anthracene. *Appl. Phys. Lett.* **85**, 3301–3303 (2004).

50. C. L. Wang, H. L. Dong, L. Jiang, W. P. Hu, Organic semiconductor crystals. *Chem. Soc. Rev.* **47**, 422–500 (2018).

51. R. K. Hallani, V. Fallah Hamidabadi, A. J. Huckaba, G. Galliani, A. Babaei, M.-G. La-Placa, A. Bahari, I. McCulloch, M. K. Nazeeruddin, M. Sessolo, H. J. Bolink, A new cross-linkable 9,10-diphenylanthracene derivative as a wide bandgap host for solution-processed organic light-emitting diodes. *J. Mater. Chem. C* **6**, 12948–12954 (2018).

52. J. Xin, Z. Li, Y. Liu, D. Liu, F. Zhu, Y. Wang, D. Yan, High-efficiency non-doped deep-blue fluorescent organic light-emitting diodes based on carbazole/phenanthroimidazole derivatives. *J. Mater. Chem. C* **8**, 10185–10190 (2020).

53. S. Hotta, H. Kimura, S. A. Lee, T. Tamaki, Synthesis of thiophene/phenylene co-oligomers. II [1]. Block and alternating co-oligomers. *J. Heterocycl. Chem.* **37**, 281–286 (2000).

54. K. Kim, M. Hua, D. Liu, J. Kim, K. J. Chen, Z. Ma, Efficiency enhancement of InGaN/GaN blue light-emitting diodes with top surface deposition of AlN/Al_2O_3. *Nano Energy* **43**, 259–269 (2018).

55. Z. F. Li, G. Y. Gan, Z. T. Ling, K. P. Guo, C. F. Si, X. Lv, H. Wang, B. Wei, Y. Y. Hao, Easily available, low-cost 9,9′-bianthracene derivatives as efficient blue hosts and deep-blue emitters in OLEDs. *Org. Electron.* **66,** 24–31 (2019).

56. J. Liu, Deep-blue efficient OLED based on NPB with little efficiency roll-off under high current density. *Appl. Phys. A* **123**:191, (2017).

57. D. H. Ahn, J. H. Jeong, J. Song, J. Y. Lee, J. H. Kwon, Highly efficient deep blue fluorescent organic light-emitting diodes boosted by thermally activated delayed fluorescence sensitization. *ACS Appl. Mater. Interfaces* **10**, 10246–10253 (2018).

58. H. Sasabe, J. Takamatsu, T. Motoyama, S. Watanabe, G. Wagenblast, N. Langer, O. Molt, E. Fuchs, C. Lennartz, J. Kido, High-efficiency blue and white organic light-emitting devices incorporating a blue iridium carbene complex. *Adv. Mater.* **22**, 5003–5007 (2010).

59. K. Y. Lu, H. H. Chou, C. H. Hsieh, Y. H. Yang, H. R. Tsai, H. Y. Tsai, L. C. Hsu, C. Y. Chen, I. C. Chen, C. H. Cheng, Wide-range color tuning of iridium biscarbene complexes from blue to red by different $N^{\wedge}N$ ligands: An alternative route for adjusting the emission colors. *Adv. Mater.* **23**, 4933–4937 (2011).

60. I. S. Park, S. Y. Lee, C. Adachi, T. Yasuda, Full-color delayed fluorescence materials based on wedge-shaped phthalonitriles and dicyanopyrazines: Systematic design, tunable photophysical properties, and OLED performance. *Adv. Funct. Mater.* **26**, 1813–1821 (2016).

61. M. Kim, S. K. Jeon, S. H. Hwang, J. Y. Lee, Stable blue thermally activated delayed fluorescent organic light-emitting diodes with three times longer lifetime than phosphorescent organic light-emitting diodes. *Adv. Mater.* **27**, 2515–2520 (2015).

62. I. Lee, J. Y. Lee, Molecular design of deep blue fluorescent emitters with 20% external quantum efficiency and narrow emission spectrum. *Org. Electron.* **29**, 160–164 (2016).

63. Q. Zhang, B. Li, S. Huang, H. Nomura, H. Tanaka, C. Adachi, Efficient blue organic light-emitting diodes employing thermally activated delayed fluorescence. *Nat. Photonics* **8**, 326–332 (2014).

64. J. W. Sun, J. Y. Baek, K.-H. Kim, C.-K. Moon, J.-H. Lee, S.-K. Kwon, Y.-H. Kim, J.-J. Kim, Thermally activated delayed fluorescence from azasiline based intramolecular charge-transfer emitter (DTPDDA) and a highly efficient blue light emitting diode. *Chem. Mater.* **27**, 6675–6681 (2015).

65. M. Kim, S. K. Jeon, S.-H. Hwang, S.-S. Lee, E. Yu, J. Y. Lee, Correlation of molecular structure with photophysical properties and device performances of thermally activated delayed fluorescent emitters. *J. Phys. Chem. C* **120**, 2485–2493 (2016).

66. X. Ouyang, X.-L. Li, X. Zhang, A. Islam, Z. Ge, S.-J. Su, Effective management of intramolecular charge transfer to obtain from blue to violet-blue OLEDs based on a couple of phenanthrene isomers. *Dyes Pigm.* **122**, 264–271 (2015).

67. S. Zhang, L. Yao, Q. Peng, W. Li, Y. Pan, R. Xiao, Y. Gao, C. Gu, Z. Wang, P. Lu, F. Li, S. Su, B. Yang, Y. Ma, Achieving a significantly increased efficiency in nondoped pure blue fluorescent OLED: A quasi-equivalent hybridized excited state. *Adv. Funct. Mater.* **25**, 1755–1762 (2015).

68. W. Li, D. Liu, F. Shen, D. Ma, Z. Wang, T. Feng, Y. Xu, B. Yang, Y. Ma, A twisting donor-acceptor molecule with an intercrossed excited state for highly efficient, deep-blue electroluminescence. *Adv. Funct. Mater.* **22**, 2797–2803 (2012).

69. H. Liu, Q. Bai, W. Li, Y. Guo, L. Yao, Y. Gao, J. Li, P. Lu, B. Yang, Y. Ma, Efficient deep-blue non-doped organic light-emitting diode with improved roll-off of efficiency based on hybrid local and charge-transfer excited state. *RSC Adv.* **6**, 70085–70090 (2016).

70. P. Y. Chou, H. H. Chou, Y. H. Chen, T. H. Su, C. Y. Liao, H. W. Lin, W. C. Lin, H. Y. Yen, I. C. Chen, C. H. Cheng, Efficient delayed fluorescence via triplet-triplet annihilation for deep-blue electroluminescence. *Chem. Commun.* **50**, 6869–6871 (2014).

71. W. Liu, S. Ying, R. Guo, X. Qiao, P. Leng, Q. Zhang, Y. Wang, D. Ma, L. Wang, Nondoped blue fluorescent organic light-emitting diodes based on benzonitrile-anthracene derivative with 10.06% external quantum efficiency and low efficiency roll-off. *J. Mater. Chem. C* **7**, 1014–1021 (2019).
